# An efficient computational scheme for solving coupled time-fractional Schrödinger equation via cubic B-spline functions

**DOI:** 10.1371/journal.pone.0296909

**Published:** 2024-05-16

**Authors:** Afzaal Mubashir Hayat, Muhammad Abbas, Homan Emadifar, Ahmed S. M. Alzaidi, Tahir Nazir, Farah Aini Abdullah

**Affiliations:** 1 Department of Mathematics, University of Sargodha, Sargodha, Pakistan; 2 Department of Mathematics, Saveetha School of Engineering, Saveetha Institute of Medical and Technical Sciences, Chennai, Tamil Nadu, India; 3 MEU Research Unit, Middle East University, Amman, Jordan; 4 Department of Mathematics, Hamedan Branch, Islamic Azad University, Hamedan, Iran; 5 Department of Mathematics and Statistics, College of Science, Taif University, Taif, Saudi Arabia; 6 School of Mathematical Sciences, Universiti Sains Malaysia, Penang, Malaysia; Qujing Normal University, CHINA

## Abstract

The time fractional Schrödinger equation contributes to our understanding of complex quantum systems, anomalous diffusion processes, and the application of fractional calculus in physics and cubic B-spline is a versatile tool in numerical analysis and computer graphics. This paper introduces a numerical method for solving the time fractional Schrödinger equation using B-spline functions and the Atangana-Baleanu fractional derivative. The proposed method employs a finite difference scheme to discretize the fractional derivative in time, while a *θ*-weighted scheme is used to discretize the space directions. The efficiency of the method is demonstrated through numerical results, and error norms are examined at various values of the non-integer parameter, temporal directions, and spatial directions.

## 1 Introduction

The significance of fractional calculus lies in its ability to provide a more accurate and comprehensive mathematical framework for modeling, analyzing, and understanding complex systems and phenomena. It bridges the gap between classical calculus and real-world complexities, opening new avenues for scientific exploration, technological advancements, and practical applications in various disciplines. Fractional calculus has undergone significant advancements to address the limitations of traditional operators. Researchers have proposed new operators or modifications to existing operators to overcome these issues. The operator developed by Caputo and Fabrizio [1] is nonsingular, which resolves the singularity problem associated with traditional operators. However, the Caputo-Fabrizio operator still exhibits non-locality, which can pose challenges in certain applications where localised behaviour is desirable. To address both the nonlocality and singularity issues, Atangana and Baleanu [2] introduced a new operator. This operator offers a valuable solution as it not only avoids singularities but also mitigates the nonlocality problem.

There are several implications for fractional derivatives in the areas of physics, mechanics, engineering, and biology [3]. In recent developments, with the use of fractional derivatives, the financial [4] and economic processes [5] are described. Many interpretations of fractional derivatives exist, such as the geometric approach [6], the informatic interpretation [7], and the economic approach [8]. Applications of fractional calculus included non-Newtonian fluid dynamics [9], rheology [10], hysteretic phenomena [11], and abnormal diffusion [12]. Studying the analytical or numerical approaches to fractional differential equations (FDEs) is extremely important since the majority of these issues may be stated as FDEs. The cubic B-spline adaptability, flexibility, and local control allow it to be appropriate for solving patrilineal differential equations [13].

The time fractional derivative was introduced into the Schrödinger equation by Naber [14]. The Schrödinger is a first-order partial differential equation with respect to time. Schrödinger’s equation can be used to describe a wide range of phenomena, from medical imaging to chemical engineering to astrophysics. The previous Zeeman–-Lorentz triplet can be found using Schrödinger’s equation. This demonstrates the wide range of uses of this equation for the right interpretation of several physical phenomena [15]. The Schrödinger equation has physical applications in atomic physics, molecular chemistry, solid state physics, quantum mechanics in nanostructures, particle physics, quantum information and computing, semiconductor physics, and nuclear physics [16]. Solutions of Schrödinger’s equation represent the probability distribution of independent particles and how likely they are to be found on a position or momentum basis [17].

The time fractional Schrödinger partial differential equation is considered as

$$\begin{array}{c} \dot{\iota }\frac{\partial^{\gamma }G\left(\xi ,t\right)}{\partial t^{\gamma }}+\frac{\partial^{2}G\left(\xi ,t\right)}{\partial \xi^{2}}+{\left|G\left(\xi ,t\right)\right|}^{2}G\left(\xi ,t\right)=U\left(\xi ,t\right),\;\;a\leq \xi \leq b,\;\;t\in \left[t_{0},T\right], \end{array}$$ (1)

with the initial condition (IC)

$$\begin{array}{c} G\left(\xi ,t_{0}\right)=g\left(\xi \right), \end{array}$$ (2)

and boundary conditions (BCs)

$$\begin{array}{c} G(a,t)=\Omega (t),\;\;G(b,t)=\Lambda (t), \end{array}$$ (3)

where 0 < *γ* < 1, and *U*(*ξ*, *t*) is the source function. The $\frac{\partial^{\gamma }}{\partial t^{\gamma }}G\left(\xi ,t\right)$ is taken in the sense of Atangana-Baleanu time fractional derivative (ABTFD).

Different numerical schemes have been used to investigate different kinds of fractional partial differential equations. The approximate solution of time fractional Schrödinger equation (TFSE) found by Zhang *et al*. [18] by proposing a fully discrete scheme using the L1 scheme based on graded mesh discretization of temporal Caputo derivative and spatial method discretization for TFSE with initial singularity. Using a non-polynomial spline, the TFSE solved by Li *et al*. [19]. A new scheme based on kernel theory and the collocation method proposed by Liu and Jiang [20] to solve TFSE. Atangana and cloot [21] solved the TFSE by using the Crank-Nicolson scheme. An implicit fully discrete local discontinuous scheme was used to solve TFSE by Wei *et al*. [22]. Heydari and Atangana [23] used the operational matrix method based on the shifted Legendre cardinal function to solve TFSE. Erfanian *et al*. [24] applied cubic B-spline (CBS) based on the finite difference formula to solve TFSE. Double Laplace transform was used by [25] to find an analytic solution for non-linear TFSE, also, [26] used the homotopy analysis transform method. A trigonometric B-spline collocation method was used by Hadhoud *et al*. [27] to solve the TFSE. A second-order accuracy difference scheme was proposed by [28] to solve TFSE. Abdeljawad [29] was found a fractional difference operators by using discrete generalized Mittag-Leffler kernels. A fractional operators with generalized Mittag-Leffler kernels and their iterated differintegrals introduced by Abdeljawad [30].

The format of this paper is as follows: The ABTFD and CBSFs are presented in Section 2. Section 3 shows the newly developed scheme. Sections 4 display stability. The effectiveness and validity of the suggested technique are examined in Section 5 and finally, Section 6 summarises the conclusion.

## 2 Preliminaries

**Definition 1**. The ABTFD $\frac{\partial^{\gamma }}{\partial t^{\gamma }}G\left(\xi ,t\right)$ of order *γ* ∈ (0, 1) is presented by [2] as

$$\begin{array}{c} \frac{\partial^{\gamma }}{\partial t^{\gamma }}G\left(\xi ,t\right)=\frac{AB(\gamma )}{1-\gamma }\int_{0}^{t}\frac{\partial }{\partial \upsilon }G\left(\xi ,\upsilon \right)E_{\gamma }[-\frac{\gamma }{1-\gamma }{\left(t-\upsilon \right)}^{\gamma }]d\upsilon , \end{array}$$ (4)

where *AB*(*γ*) is a normalized form of function that has the property *AB*(*γ* = 0) = *AB*(*γ* = 1) = 1. *E*_*γ*,*σ*_(*ξ*) is the MLF with *E*_*γ*,1_(*ξ*) = *E*_*γ*_(*ξ*), that is defined as:

$$\begin{array}{c} E_{\gamma ,\sigma }\left(\xi \right)=\sum_{y=0}^{\infty }\frac{\xi^{y}}{\Gamma (\gamma y+\sigma )}. \end{array}$$

Some properties of MLF are as following by fixing *γ* and *σ*,

- *E*_1,1_(*ξ*) = *e*^*ξ*^,
-
$E_{1,2}\left(\xi \right)=\frac{e^{\xi }-1}{\xi },$

- *E*_2,1_(*ξ*^2^) = cosh(*ξ*),
- *E*_2,1_(−*ξ*^2^) = cos(*ξ*).

To decompose the complex function *G*(*ξ*, *t*) into real and imaginary parts $\hat{\psi }\left(\xi ,t\right)$ and $\hat{\phi }\left(\xi ,t\right)$ respectively

$$\begin{array}{c} G\left(\xi ,t\right)=\hat{\psi }\left(\xi ,t\right)+\dot{\iota }\hat{\phi }\left(\xi ,t\right) \end{array}$$ (5)

Using (5) into (1) results in coupled system of nonlinear partial differential equations as

$$\begin{array}{c} \frac{\partial^{\gamma }\hat{\phi }\left(\xi ,t\right)}{\partial t^{\gamma }}-\frac{\partial^{2}\hat{\psi }\left(\xi ,t\right)}{\partial \xi^{2}}-\left({\hat{\psi }}^{2}+{\hat{\phi }}^{2}\right)\hat{\psi }=-U_{Re}\left(\xi ,t\right) \end{array}$$ (6)

$$\begin{array}{c} \frac{\partial^{\gamma }\hat{\psi }\left(\xi ,t\right)}{\partial t^{\gamma }}+\frac{\partial^{2}\hat{\phi }\left(\xi ,t\right)}{\partial \xi^{2}}+\left({\hat{\psi }}^{2}+{\hat{\phi }}^{2}\right)\hat{\phi }=U_{Im}\left(\xi ,t\right) \end{array}$$ (7)

where *U*_*Re*_(*ξ*, *t*) is real and *U*_*Im*_(*ξ*, *t*) is imaginary parts of *U*(*ξ*, *t*). Moreover, the initial conditions of (1) as follows:

$$\begin{array}{c} \hat{\psi }\left(\xi ,0\right)=g_{Re}\left(\xi \right),\;\hat{\phi }\left(\xi ,0\right)=g_{Im}\left(\xi \right),\;a\leq \xi \leq b, \end{array}$$ (8)

where *g*_*Re*_(*ξ*) and *g*_*Im*_(*ξ*) are real and imaginary parts of *g*(*ξ*), respectively, and boundary conditions as

$$\begin{array}{c} \hat{\psi }\left(a,t\right)=\Omega_{Re}\left(t\right),\;\hat{\psi }\left(b,t\right)=\Lambda_{Re}\left(t\right),\;\hat{\phi }\left(a,t\right)=\Omega_{Im}\left(t\right),\;\hat{\phi }\left(b,t\right)=\Lambda_{Im}\left(t\right),\;t\geq 0, \end{array}$$ (9)

where Ω_*Re*_(*t*) and Ω_*Im*_(*t*) are real and imaginary parts of Ω(*t*), respectively, and Λ_*Re*_(*t*) and Λ_*Im*_(*t*) are real and imaginary parts of Λ(*t*), respectively.

### 2.1 Cubic B-spline basis functions

The spatial domain [*a*, *b*] be divided into equal length of *N* subintervals with $h=\frac{b-a}{N}$ such that {*a* = *ξ*_0_, *ξ*_1_ ⋯, *ξ*_*N*_ = *b*} with *ξ*_*r*_ < *ξ*_*r*+1_, where *ξ*_*r*_ = *hk* + *ξ*_0_, *r* = 0(1)*N*.

Now, let *ϕ*(*ξ*, *t*) be the CBSFs approach for $\hat{\phi }\left(\xi ,t\right)$ and *ψ*(*ξ*, *t*) be the CBSFs approach for $\hat{\psi }\left(\xi ,t\right)$

$$\begin{array}{c} \phi \left(\xi ,t\right)=\sum_{r=-1}^{N+1}\Psi_{r}^{m}\left(t\right){\hat{C}}_{r}\left(\xi \right),\;\psi \left(\xi ,t\right)=\sum_{r=-1}^{N+1}\Phi_{r}^{m}\left(t\right){\hat{C}}_{r}\left(\xi \right), \end{array}$$ (10)

where control points $\Psi_{r}^{m}\left(t\right)$ and $\Phi_{r}^{m}\left(t\right)$ to be calculated at every temporal stage and CBSFs are defined as:

$$\begin{array}{c} {\hat{C}}_{r}\left(\xi \right)=\frac{1}{6h^{3}}\{\begin{array}{cc} {\left(\xi -\xi_{r-2}\right)}^{3}, & \text{if}\;\xi \in [\xi_{r-2},\xi_{r-1}),\\ 3\left(\xi -\xi_{r-1}\right)h^{2}+3{\left(\xi -\xi_{r-1}\right)}^{2}h-3{\left(\xi -\xi_{r-1}\right)}^{3}+h^{3}, & \text{if}\;\xi \in [\xi_{r-1},\xi_{r}),\\ h^{3}+3h^{2}\left(\xi_{r+1}-\xi \right)+3h{\left(\xi_{r+1}-\xi \right)}^{2}-3{\left(\xi_{r+1}-\xi \right)}^{3}, & \text{if}\;\xi \in [\xi_{r},\xi_{r+1}),\\ {\left(\xi_{r+2}-\xi \right)}^{3}, & \text{if}\;\xi \in [\xi_{r+1},\xi_{r+2}),\\ 0, & \text{otherwise}. \end{array} \end{array}$$ (11)

Numerous geometrical features, including the geometric invariability, symmetry, the convex hull characteristic, local support, non-negativity, and the partition of unity, are preserved in the CBSFs [31]. Additionally, ${\hat{C}}_{-1},{\hat{C}}_{0}\cdots ,{\hat{C}}_{N+1}$ have been constructed. The Eq (10) and the Eq (11) gives the following approximations:

$$\begin{array}{c} \left\{ \begin{array}{c} {\left(\phi \right)}_{r}^{m}=\left(\frac{1}{6}\right)\Psi_{r-1}^{m}+\left(\frac{4}{6}\right)\Psi_{r}^{m}+\left(\frac{1}{6}\right)\Psi_{r+1}^{m},\\ {\left(\phi_{\xi }\right)}_{r}^{m}=\left(\frac{1}{2h}\right)\Psi_{r+1}^{m}+\left(-\frac{1}{2h}\right)\Psi_{r-1}^{m},\\ {\left(\phi_{\xi \xi }\right)}_{r}^{m}=\left(\frac{1}{h^{2}}\right)\Psi_{r-1}^{m}+\left(-\frac{2}{h^{2}}\right)\Psi_{r}^{m}+\left(\frac{1}{h^{2}}\right)\Psi_{r+1}^{m}. \end{array}\right. \end{array}$$ (12)

$$\begin{array}{c} \left\{ \begin{array}{c} {\left(\psi \right)}_{r}^{m}=\left(\frac{1}{6}\right)\Phi_{r-1}^{m}+\left(\frac{4}{6}\right)\Phi_{r}^{m}+\left(\frac{1}{6}\right)\Phi_{r+1}^{m},\\ {\left(\psi_{\xi }\right)}_{r}^{m}=\left(\frac{1}{2h}\right)\Phi_{r+1}^{m}+\left(-\frac{1}{2h}\right)\Phi_{r-1}^{m},\\ {\left(\psi_{\xi \xi }\right)}_{r}^{m}=\left(\frac{1}{h^{2}}\right)\Phi_{r-1}^{m}+\left(-\frac{2}{h^{2}}\right)\Phi_{r}^{m}+\left(\frac{1}{h^{2}}\right)\Phi_{r+1}^{m}. \end{array}\right. \end{array}$$ (13)

## 3 Illustration of the scheme

Suppose [0, *T*] the time domain be divided into *M* subintervals of equal length with $\Delta t=\frac{T}{M}$ using {0 = *t*_0_, *t*_1_ ⋯, *t*_*M*_ = *T*} with *t*_*m*_ < *t*_*m*+1_ where *t*_*m*_ = *m*Δ*t* and *m* = 0:1:*M*. The ABTFD in (1) is discretized at *t* = *t*_*m*+1_ as

$$\begin{array}{ccc} \frac{\partial^{\gamma }}{\partial t^{\gamma }}G\left(\xi ,t_{m+1}\right) & = & \frac{AB(\gamma )}{1-\gamma }\int_{0}^{t_{m+1}}\frac{\partial }{\partial \upsilon }G\left(\xi ,\upsilon \right)E_{\gamma }[-\frac{\gamma }{1-\gamma }{\left(t_{m+1}-\upsilon \right)}^{\gamma }]d\upsilon ,\;\;\;\;\;0<\gamma <1,\\ & = & \frac{AB(\gamma )}{1-\gamma }\sum_{v=0}^{m}\int_{t_{v}}^{t_{v+1}}\frac{\partial }{\partial \upsilon }G\left(\xi ,\upsilon \right)E_{\gamma }[-\frac{\gamma }{1-\gamma }{\left(t_{m+1}-\upsilon \right)}^{\gamma }]d\upsilon . \end{array}$$ (14)

Utilizing forward difference formulation, the Eq (14) becomes

$$\begin{array}{cc} \frac{\partial^{\gamma }}{\partial t^{\gamma }}G\left(\xi ,t_{m+1}\right) & =\frac{AB(\gamma )}{1-\gamma }\sum_{v=0}^{m}\frac{G\left(\xi ,t_{v+1}\right)-G\left(\xi ,t_{v}\right)}{\Delta t}\int_{t_{v}}^{t_{v+1}}E_{\gamma }[-\frac{\gamma }{1-\gamma }{\left(t_{m+1}-\upsilon \right)}^{\gamma }]d\upsilon +\lambda_{\Delta t}^{m+1}\\ & =\frac{AB(\gamma )}{1-\gamma }\sum_{v=0}^{m}[G\left(\xi ,t_{m-v+1}\right)-G\left(\xi ,t_{m-v}\right)]\{\left(v+1\right)E_{\gamma ,2}[-\frac{\gamma }{1-\gamma }((v+1)\Delta t{)}^{\gamma }]\\ & -vE_{\gamma ,2}[-\frac{\gamma }{1-\gamma }{\left(v\Delta t\right)}^{\gamma }]\}+\lambda_{\Delta t}^{m+1}\\ & =\frac{AB(\gamma )}{1-\gamma }\sum_{v=0}^{m}[G\left(\xi ,t_{m-v+1}\right)-G\left(\xi ,t_{m-v}\right)](\left(v+1\right)E_{v+1}-vE_{v})+\lambda_{\Delta t}^{m+1}. \end{array}$$

Hence

$$\begin{array}{c} \frac{\partial^{\gamma }}{\partial t^{\gamma }}G\left(\xi ,t_{m+1}\right)=\frac{AB(\gamma )}{1-\gamma }\sum_{v=0}^{m}w_{v}[G\left(\xi ,t_{m-v+1}\right)-G\left(\xi ,t_{m-v}\right)]+\lambda_{\Delta t}^{m+1}, \end{array}$$ (15)

where $E_{v}=E_{\gamma ,2}[-\frac{\gamma }{1-\gamma }{\left(v\Delta t\right)}^{\gamma }]$ and *w*_*v*_ = (*v* + 1)*E*_*v*+1_ − *vE*_*v*_. Simple observation reveals that

- *w*_*v*_ > 0 and *w*_0_ = *E*_1_, *v* = 0:1:*m*,
- *w*_0_ > *w*_1_ > *w*_2_ > … > *w*_*v*_, *w*_*v*_ → 0 as *v* → ∞,
-
$\sum_{v=0}^{m}\left(w_{v}-w_{v+1}\right)+w_{m+1}=\left(E_{1}-w_{1}\right)+\sum_{v=1}^{m-1}\left(w_{v}-w_{v+1}\right)+w_{m}=E_{1}$ .

Also, the truncation error $\lambda_{\Delta t}^{m+1}$ is shown in [31] as

$$\begin{array}{cc} \lambda_{\Delta t}^{m+1} & =\frac{AB(\gamma )}{1-\gamma }\sum_{v=0}^{m}\int_{t_{v}}^{t_{v+1}}\frac{\Delta t}{2}\frac{\partial^{2}G\left(\xi ,t_{v}\right)}{\partial t^{2}}E_{\gamma }[-\frac{\gamma }{1-\gamma }{\left(t_{m+1}-\upsilon \right)}^{\gamma }]d\upsilon \\ & =\frac{AB(\gamma )}{1-\gamma }\frac{{\left(\Delta t\right)}^{2}}{2}\sum_{v=0}^{m}\frac{\partial^{2}G\left(\xi ,t_{v}\right)}{\partial t^{2}}\{\left(m-v+1\right)E_{\gamma ,2}[-\frac{\gamma }{1-\gamma }((m-v+1)\Delta t{)}^{\gamma }]\\ & -\left(m-v\right)E_{\gamma ,2}[-\frac{\gamma }{1-\gamma }{\left(\left(m-v\right)\Delta t\right)}^{\gamma }]\}\\ & =\frac{AB(\gamma )}{1-\gamma }\frac{{\left(\Delta t\right)}^{2}}{2}\sum_{v=0}^{m}\frac{\partial^{2}G\left(\xi ,t_{v}\right)}{\partial t^{2}}(\left(m-v+1\right)E_{m-v+1}-\left(m-v\right)E_{m-v})\\ & \leq \frac{AB(\gamma )}{1-\gamma }\frac{{\left(\Delta t\right)}^{2}}{2}[\underset{0\leq t\leq t_{m}}{\text{max}}\frac{\partial^{2}G\left(\xi ,t\right)}{\partial t^{2}}]c_{1}, \end{array}$$

where *c*_1_ is constant.

$$\begin{array}{c} |\lambda_{\Delta t}^{m+1}{|\leq \vartheta \left(\Delta t\right)}^{2}, \end{array}$$ (16)

where Ψ is constant.

Now using *θ*-weighted scheme and (15) the Eqs (6) and (7) becomes

$$\begin{array}{c} \frac{AB(\gamma )}{1-\gamma }\sum_{v=0}^{m}w_{v}[\phi \left(\xi ,t_{m-v+1}\right)-\phi \left(\xi ,t_{m-v}\right)]=\theta [\psi_{\xi \xi }\left(\xi ,t_{m+1}\right)+(\psi^{2}\left(\xi ,t_{m+1}\right)\\ +\phi^{2}\left(\xi ,t_{m+1}\right))\psi (\xi ,t_{m+1}]+\left(1-\theta \right)[\psi_{\xi \xi }\left(\xi ,t_{m}\right)+(\psi^{2}\left(\xi ,t_{m}\right)\\ +\phi^{2}\left(\xi ,t_{m}\right))\psi (\xi ,t_{m}]-U_{Re}\left(\xi ,t_{m+1}\right). \end{array}$$ (17)

$$\begin{array}{c} \frac{AB(\gamma )}{1-\gamma }\sum_{v=0}^{m}w_{v}[\psi \left(\xi ,t_{m-v+1}\right)-\psi \left(z,t_{m-v}\right)]=-\theta [\phi_{\xi \xi }\left(\xi ,t_{m+1}\right)+(\psi^{2}\left(\xi ,t_{m+1}\right)\\ +\phi^{2}\left(\xi ,t_{m+1}\right))\phi (\xi ,t_{m+1}]-\left(1-\theta \right)[\phi_{\xi \xi }\left(\xi ,t_{m}\right)+(\psi^{2}\left(\xi ,t_{m}\right)\\ +\phi^{2}\left(\xi ,t_{m}\right))\phi (\xi ,t_{m}]+U_{Re}\left(\xi ,t_{m+1}\right). \end{array}$$ (18)

Discretizing (17) and (18) along spatial direction for *θ* = 1 and using the linearization formula defined in [27] as

$$\begin{array}{c} {\left(\psi^{s}\right)}_{r}^{m+1}=s{\left(\psi \right)}_{r}^{m+1}{\left(\psi^{s-1}\right)}_{r}^{m}-\left(s-1\right){\left(\psi^{s}\right)}_{r}^{m}. \end{array}$$ (19)

$$\begin{array}{c} {\left(\psi \phi \right)}_{r}^{m+1}=\phi_{r}^{m+1}\psi_{r}^{m}+\phi_{r}^{m}\psi_{r}^{m+1}-\phi_{r}^{m}\psi_{r}^{m} \end{array}$$ (20)

we have

$$\begin{array}{c} z_{1}\phi_{r}^{m+1}-\eta {\left(\psi_{\xi \xi }\right)}_{r}^{m+1}+z_{2}\psi_{r}^{m+1}\\ \;=E_{1}\phi_{r}^{m}-z_{5}\psi_{r}^{m}-\sum_{v=1}^{m}w_{v}\left(\phi_{r}^{m-v+1}-\phi_{r}^{m-v}\right)-\eta {\left(U_{Re}\right)}_{r}^{m+1}, \end{array}$$ (21)

$$\begin{array}{c} z_{3}\psi_{r}^{m+1}+\eta {\left(\phi_{\xi \xi }\right)}_{r}^{m+1}+z_{4}\phi_{r}^{m+1}\\ \;=E_{1}\psi_{r}^{m}+z_{5}\phi_{r}^{m}-\sum_{v=1}^{m}w_{v}\left(\psi_{r}^{m-v+1}-\psi_{r}^{m-v}\right)-\eta {\left(U_{Im}\right)}_{r}^{m+1}, \end{array}$$ (22)

where $\eta =\frac{1-\gamma }{AB(\gamma )}$, $\psi_{r}^{m}=\psi \left(\xi_{r},t_{m}\right)$, $\phi_{r}^{m}=\phi \left(\xi_{r},t_{m}\right)$, $\xi_{1}=E_{1}-2\eta \phi_{r}^{m}\psi_{r}^{m}$, $z_{2}=-\eta \left({\left(\phi_{r}^{m}\right)}^{2}+3{\left(\psi_{r}^{m}\right)}^{2}\right)$, $z_{3}=E_{1}+2\eta \phi_{r}^{m}\psi_{r}^{m}$, $z_{4}=\eta \left(3{\left(\phi_{r}^{m}\right)}^{2}+{\left(\psi_{r}^{m}\right)}^{2}\right)$, $z_{5}=2\eta {\left(\psi^{2}\right)}_{r}^{m}+2\eta {\left(\phi^{2}\right)}_{r}^{m}$, ${\left(U_{Im}\right)}_{r}^{m+1}=U_{Im}\left(\xi_{r},t_{m+1}\right)$ and ${\left(U_{Re}\right)}_{r}^{m+1}=U_{Re}\left(\xi_{r},t_{m+1}\right)$.

Using (12), (13) in (21) and (22), we get

$$\begin{array}{c} z_{1}\left[\frac{1}{6}\Psi_{r-1}^{m+1}+\frac{4}{6}\Psi_{r}^{m+1}+\frac{1}{6}\Psi_{r+1}^{m+1}\right]-\eta \left[\frac{1}{h^{2}}\Phi_{r-1}^{m+1}-\frac{2}{h^{2}}\Phi_{r}^{m+1}+\frac{1}{h^{2}}\Phi_{r+1}^{m+1}\right]\\ +z_{2}\left[\frac{1}{6}\Phi_{r-1}^{m+1}+\frac{4}{6}\Phi_{r}^{m+1}+\frac{1}{6}\Phi_{r+1}^{m+1}\right]=E_{1}\left[\frac{1}{6}\Psi_{r-1}^{m}+\frac{4}{6}\Psi_{r}^{m}+\frac{1}{6}\Psi_{r+1}^{m}\right]\\ -z_{5}\left[\frac{1}{6}\Phi_{r-1}^{m}+\frac{4}{6}\Phi_{r}^{m}+\frac{1}{6}\Phi_{r+1}^{m}\right]-\sum_{v=1}^{m}w_{v}[\frac{1}{6}\left(\Psi_{r-1}^{m-v+1}-\Psi_{r-1}^{m-v}\right)\\ +\frac{4}{6}\left(\Psi_{r}^{m-v+1}-\Psi_{r}^{m-v}\right)+\frac{1}{6}\left(\Psi_{r+1}^{m-v+1}-\Psi_{r+1}^{m-v}\right)]+\eta \;\;{\left(U_{Re}\right)}_{r}^{m+1}, \end{array}$$ (23)

$$\begin{array}{c} z_{3}\left[\frac{1}{6}\Phi_{r-1}^{m+1}+\frac{4}{6}\Phi_{r}^{m+1}+\frac{1}{6}\Phi_{r+1}^{m+1}\right]+\eta \left[\frac{1}{h^{2}}\Psi_{r-1}^{m+1}-\frac{2}{h^{2}}\Psi_{r}^{m+1}+\frac{1}{h^{2}}\Psi_{r+1}^{m+1}\right]\\ +z_{4}\left[\frac{1}{6}\Psi_{r-1}^{m+1}+\frac{4}{6}\Psi_{r}^{m+1}+\frac{1}{6}\Psi_{r+1}^{m+1}\right]=E_{1}\left[\frac{1}{6}\Phi_{r-1}^{m}+\frac{4}{6}\Phi_{r}^{m}+\frac{1}{6}\Phi_{r+1}^{m}\right]\\ +z_{5}\left[\frac{1}{6}\Psi_{r-1}^{m}+\frac{4}{6}\Psi_{r}^{m}+\frac{1}{6}\Psi_{r+1}^{m}\right]-\sum_{v=1}^{m}w_{v}[\frac{1}{6}\left(\Phi_{r-1}^{m-v+1}-\Phi_{r-1}^{m-v}\right)\\ +\frac{4}{6}\left(\Phi_{r}^{m-v+1}-\Phi_{r}^{m-v}\right)+\frac{1}{6}\left(\Phi_{r+1}^{m-v+1}-\Phi_{r+1}^{m-v}\right)]+\eta \;\;{\left(U_{Im}\right)}_{r}^{m+1}, \end{array}$$ (24)

Hence

$$\begin{array}{c} z_{1}\left[\frac{1}{6}\Psi_{r-1}^{m+1}+\frac{4}{6}\Psi_{r}^{m+1}+\frac{1}{6}\Psi_{r+1}^{m+1}\right]+b_{1}\Phi_{r-1}^{m+1}+b_{2}\Phi_{r}^{m+1}+b_{1}\Phi_{r+1}^{m+1}\\ =E_{1}\left[\frac{1}{6}\Psi_{r-1}^{m}+\frac{4}{6}\Psi_{r}^{m}+\frac{1}{6}\Psi_{r+1}^{m}\right]-z_{5}\left[\frac{1}{6}\Phi_{r-1}^{m}+\frac{4}{6}\Phi_{r}^{m}+\frac{1}{6}\Phi_{r+1}^{m}\right]\\ -\sum_{v=1}^{m}w_{v}\left[\frac{1}{6}\left(\Psi_{r-1}^{m-v+1}-\Psi_{r-1}^{m-v}\right)+\frac{4}{6}\left(\Psi_{r}^{m-v+1}-\Psi_{r}^{m-v}\right)+\frac{1}{6}\left(\Psi_{r+1}^{m-v+1}-\Psi_{r+1}^{m-v}\right)\right]-\eta \;\;{\left(U_{Re}\right)}_{r}^{m+1}, \end{array}$$ (25)

$$\begin{array}{c} z_{3}\left[\frac{1}{6}\Phi_{r-1}^{m+1}+\frac{4}{6}\Phi_{r}^{m+1}+\frac{1}{6}\Phi_{r+1}^{m+1}\right]+b_{3}\Psi_{r-1}^{m+1}+b_{4}\Psi_{r}^{m+1}+b_{3}\Psi_{r+1}^{m+1}\\ =E_{1}\left[\frac{1}{6}\Phi_{r-1}^{m}+\frac{4}{6}\Phi_{r}^{m}+\frac{1}{6}\Phi_{r+1}^{m}\right]+z_{5}\left[\frac{1}{6}\Psi_{r-1}^{m}+\frac{4}{6}\Psi_{r}^{m}+\frac{1}{6}\Psi_{r+1}^{m}\right]\\ -\sum_{v=1}^{m}w_{v}\left[\frac{1}{6}\left(\Phi_{r-1}^{m-v+1}-\Phi_{r-1}^{m-v}\right)+\frac{4}{6}\left(\Phi_{r}^{m-v+1}-\Phi_{r}^{m-v}\right)+\frac{1}{6}\left(\Phi_{r+1}^{m-v+1}-\Phi_{r+1}^{m-v}\right)\right]+\eta \;\;{\left(U_{Im}\right)}_{r}^{m+1}, \end{array}$$ (26)

where $b_{1}=\frac{-\eta }{h^{2}}+\frac{z_{2}}{6}$, $b_{2}=\frac{2\eta }{h^{2}}+\frac{4z_{2}}{6}$, $b_{3}=\frac{\eta }{h^{2}}+\frac{z_{4}}{6}$ and $b_{4}=\frac{-2\eta }{h^{2}}+\frac{4z_{4}}{6}$. This system (25) and (26) has 2*N* + 2 linear equations with 2*N*+ 6 unknowns. Four more equations can be found for unique solution from the given BCs (9).

$$\begin{array}{c} \left\{ \begin{array}{c} \frac{1}{6}\Psi_{-1}^{m+1}+\frac{4}{6}\Psi_{0}^{m+1}+\frac{1}{6}\Psi_{1}^{m+1}={\left(\Omega_{Im}\right)}_{1}^{m+1}\\ \frac{1}{6}\Psi_{N-1}^{m+1}+\frac{4}{6}\Psi_{N}^{m+1}+\frac{1}{6}\Psi_{N+1}^{m+1}={\left(\Lambda_{Im}\right)}_{2}^{m+1} \end{array}\right. \end{array}$$ (27)

$$\begin{array}{c} \left\{ \begin{array}{c} \frac{1}{6}\Phi_{-1}^{m+1}+\frac{4}{6}\Phi_{0}^{m+1}+\frac{1}{6}\Phi_{1}^{m+1}={\left(\Omega_{Re}\right)}_{1}^{m+1}\\ \frac{1}{6}\Phi_{N-1}^{m+1}+\frac{4}{6}\Phi_{N}^{m+1}+\frac{1}{6}\Phi_{N+1}^{m+1}={\left(\Lambda_{Re}\right)}_{2}^{m+1} \end{array}\right. \end{array}$$ (28)

The initial vectors $\Psi_{r}^{0}$ and $\Phi_{r}^{0}$ are obtained by using the initial conditions (8) and their derivative as:

$$\begin{array}{c} \left\{ \begin{array}{c} {\left(\phi_{\xi }\right)}_{r}^{0}=g_{Im}^{\prime }\left(\xi_{r}\right),\\ {\left(\phi \right)}_{r}^{0}=g_{Im}\left(\xi_{r}\right),\\ {\left(\phi_{\xi }\right)}_{r}^{0}=g_{Im}^{\prime }\left(\xi_{r}\right),\\ {\left(\psi_{\xi }\right)}_{r}^{0}=g_{Re}^{\prime }\left(\xi_{r}\right),\\ {\left(\psi \right)}_{r}^{0}=g_{Re}\left(\xi_{r}\right),\\ {\left(\psi_{\xi }\right)}_{r}^{0}=g_{Re}^{\prime }\left(\xi_{r}\right), \end{array}\right. \end{array}$$ (29)

Any numerical algorithm can be used to solve the Eq (29). Mathematica 10 is used to conduct the numerical results.

## 4 The stability

The stability of the scheme (25) and (26) is analysed by using the Von Neumann method as [27]. First, we linearize the nonlinear terms *ϕ* and *ψ* as local constants *κ*_1_ and *τ*_1_, respectively, as is done in the Von Neumann method.

$\epsilon_{\Psi_{r}^{m}}=\Psi_{r}^{m+1}-{\tilde{\Psi }}_{r}^{m+1}$ , $\epsilon_{\Phi_{r}^{m}}=\Phi_{r}^{m+1}-{\tilde{\Phi }}_{r}^{m+1}$ are the approximate solution of (25) and (26) we can easily obtained round-off error equations

Taking (*U* = 0) in (25) the Eq (26) becomes

$$\begin{array}{c} z_{1}\left[\frac{1}{6}\epsilon_{\Psi_{r-1}^{m+1}}+\frac{4}{6}\epsilon_{\Psi_{r}^{m+1}}+\frac{1}{6}\epsilon_{\Psi_{r+1}^{m+1}}\right]+b_{1}\epsilon_{\Phi_{r-1}^{m+1}}+b_{2}\epsilon_{\Phi_{r}^{m+1}}+b_{1}\epsilon_{\Phi_{r+1}^{m+1}}\\ =E_{1}\left[\frac{1}{6}\epsilon_{\Psi_{r-1}^{m}}+\frac{4}{6}\epsilon_{\Psi_{r}^{m}}+\frac{1}{6}\epsilon_{\Psi_{r+1}^{m}}\right]-z_{5}\left[\frac{1}{6}\epsilon_{\Phi_{r-1}^{m}}+\frac{4}{6}\epsilon_{\Phi_{r}^{m}}+\frac{1}{6}\epsilon_{\Phi_{r+1}^{m}}\right]\\ -\sum_{v=1}^{m}w_{v}\left[\frac{1}{6}\left(\epsilon_{\Psi_{r-1}^{m-v+1}}-\epsilon_{\Psi_{r-1}^{m-v}}\right)+\frac{4}{6}\left(\epsilon_{\Psi_{r}^{m-v+1}}-\epsilon_{\Psi_{r}^{m-v}}\right)+\frac{1}{6}\left(\epsilon_{\Psi_{r+1}^{m-v+1}}-\epsilon_{\Psi_{r+1}^{m-v}}\right)\right], \end{array}$$ (30)

$$\begin{array}{c} z_{3}\left[\frac{1}{6}\epsilon_{\Phi_{r-1}^{m+1}}+\frac{4}{6}\epsilon_{\Phi_{r}^{m+1}}+\frac{1}{6}\epsilon_{\Phi_{r+1}^{m+1}}\right]+b_{3}\epsilon_{\Psi_{r-1}^{m+1}}+b_{4}\epsilon_{\Psi_{r}^{m+1}}+b_{3}\epsilon_{\Psi_{r+1}^{m+1}}\\ =E_{1}\left[\frac{1}{6}\epsilon_{\Phi_{r-1}^{m}}+\frac{4}{6}\epsilon_{\Phi_{r}^{m}}+\frac{1}{6}\epsilon_{\Phi_{r+1}^{m}}\right]+z_{5}\left[\frac{1}{6}\epsilon_{\Psi_{r-1}^{m}}+\frac{4}{6}\epsilon_{\Psi_{r}^{m}}+\frac{1}{6}\epsilon_{\Psi_{r+1}^{m}}\right]\\ -\sum_{v=1}^{m}w_{v}\left[\frac{1}{6}\left(\epsilon_{\Phi_{r-1}^{m-v+1}}-\epsilon_{\Phi_{r-1}^{m-v}}\right)+\frac{4}{6}\left(\epsilon_{\Phi_{r}^{m-v+1}}-\epsilon_{\Phi_{r}^{m-v}}\right)+\frac{1}{6}\left(\epsilon_{\Phi_{r+1}^{m-v+1}}-\epsilon_{\Phi_{r+1}^{m-v}}\right)\right], \end{array}$$ (31)

where *z*_1_ = *E*_1_ − 2*ηκ*_1_*τ*_1_, $z_{2}=-\eta \left(\kappa_{1}^{2}+3\tau_{1}^{2}\right)$, *z*_3_ = *E*_1_ + 2*ηκ*_1_*τ*_1_, $z_{4}=\eta \left(3\kappa_{1}^{2}+\tau_{1}^{2}\right)$, $z_{5}=2\eta \tau_{1}^{2}+2\eta \kappa_{1}^{2}$.

Suppose that the Eqs (30)–(33) have solutions of the form

$$\begin{array}{c} \epsilon_{\Psi_{r}^{m}}=p_{m}e^{\dot{\iota }r\alpha h},\;\epsilon_{\Phi_{r}^{m}}=q_{m}e^{\dot{\iota }r\alpha h} \end{array}$$ (32)

where $\dot{\iota }=\sqrt{-1}$ and *α* is real. Using (32) in the Eqs (30)–(33) and dividing by $e^{\dot{\iota }r\alpha h}$ and collecting the like terms, we have

$$\begin{array}{c} z_{1}p_{m+1}\left(2cos\left(\alpha h\right)+4\right)+6q_{m+1}\left(2b_{1}cos\left(\alpha h\right)+b_{2}\right)\\ =E_{1}p_{m}\left(2cos\left(\alpha h\right)+4\right)-z_{5}q_{m}\left(2cos\left(\alpha h\right)+4\right)-\sum_{v=1}^{m}w_{v}\left(p_{m-v+1}-p_{m-v}\right)\left(2cos\left(\alpha h\right)+4\right) \end{array}$$ (33)

$$\begin{array}{c} z_{3}q_{m+1}\left(2cos\left(\alpha h\right)+4\right)+6p_{m+1}\left(2b_{3}cos\left(\alpha h\right)+b_{4}\right)\\ =E_{1}q_{m}\left(2cos\left(\alpha h\right)+4\right)-z_{5}p_{m}\left(2cos\left(\alpha h\right)+4\right)-\sum_{v=1}^{m}w_{v}\left(q_{m-v+1}-q_{m-v}\right)\left(2cos\left(\alpha h\right)+4\right) \end{array}$$ (34)

From (33) and (34) we get

$$\begin{array}{c} z_{1}p_{m+1}+6q_{m+1}\gamma_{1}=E_{1}p_{m}-z_{5}q_{m}-\sum_{v=1}^{m}w_{v}\left(p_{m-v+1}-p_{m-v}\right) \end{array}$$ (35)

$$\begin{array}{c} z_{3}q_{m+1}+6p_{m+1}\gamma_{2}=E_{1}q_{m}-z_{5}p_{m}-\sum_{v=1}^{m}w_{v}\left(q_{m-v+1}-q_{m-v}\right) \end{array}$$ (36)

where *m* = 0, 1, 2, …, $\gamma_{1}=\frac{2b_{1}cos\left(\alpha h\right)+b_{2}}{2cos(\alpha h)+4}$ and $\gamma_{2}=\frac{2b_{3}cos\left(\alpha h\right)+b_{4}}{2cos(\alpha h)+4}$. Using Wolfram Mathematica to solve (35) and (36), we obtained

$$\begin{array}{c} p_{m+1}=\frac{p_{m}\left(E_{1}z_{3}-6\gamma_{1}z_{5}\right)}{z_{1}z_{3}-36\gamma_{1}\gamma_{2}}-\frac{q_{m}\left(z_{3}z_{5}+6\gamma_{1}E_{1}\right)}{z_{1}z_{3}-36\gamma_{1}\gamma_{2}}\\ \;+\frac{6\gamma_{1}\sum_{v=1}^{m}w_{v}\left(q_{m-v+1}-q_{m-v}\right)}{z_{1}z_{3}-36\gamma_{1}\gamma_{2}}-\frac{z_{3}\sum_{v=1}^{m}w_{v}\left(p_{m-v+1}-p_{m-v}\right)}{z_{1}z_{3}-36\gamma_{1}\gamma_{2}} \end{array}$$ (37)

$$\begin{array}{c} q_{m+1}=\frac{q_{m}\left(E_{1}z_{1}+6\gamma_{2}z_{5}\right)}{z_{1}z_{3}-36\gamma_{1}\gamma_{2}}+\frac{p_{m}\left(z_{1}z_{5}+6\gamma_{2}E_{1}\right)}{z_{1}z_{3}-36\gamma_{1}\gamma_{2}}\\ \;-\frac{z_{1}\sum_{v=1}^{m}w_{v}\left(q_{m-v+1}-q_{m-v}\right)}{z_{1}z_{3}-36\gamma_{1}\gamma_{2}}+\frac{6\gamma_{2}\sum_{v=1}^{m}w_{v}\left(p_{m-v+1}-p_{m-v}\right)}{z_{1}z_{3}-36\gamma_{1}\gamma_{2}} \end{array}$$ (38)

Now, using values of *z*_1_ = *E*_1_ − 2*ηκ*_1_*τ*_1_, $z_{2}=-\eta \left(\kappa_{1}^{2}+3\tau_{1}^{2}\right)$, *z*_3_ = *E*_1_ + 2*ηκ*_1_*τ*_1_, $z_{4}=\eta \left(3\kappa_{1}^{2}+\tau_{1}^{2}\right)$, $z_{5}=2\eta \tau_{1}^{2}+2\eta \kappa_{1}^{2}$ in (37) and (38)

$$\begin{array}{c} p_{m+1}=\frac{p_{m}\left(E_{1}\left(E_{1}+2\eta \kappa_{1}\tau_{1}\right)-6\gamma_{1}\left(2\eta \tau_{1}^{2}+2\eta \kappa_{1}^{2}\right)\right)}{\left(E_{1}-2\eta \kappa_{1}\tau_{1}\right)\left(E_{1}+2\eta \kappa_{1}\tau_{1}\right)-36\gamma_{1}\gamma_{2}}\\ -\frac{q_{m}\left(\left(E_{1}+2\eta \kappa_{1}\tau_{1}\right)\left(2\eta \tau_{1}^{2}+2\eta \kappa_{1}^{2}\right)+6\gamma_{1}E_{1}\right)}{\left(E_{1}-2\eta \kappa_{1}\tau_{1}\right)\left(E_{1}+2\eta \kappa_{1}\tau_{1}\right)-36\gamma_{1}\gamma_{2}}+\frac{6\gamma_{1}\sum_{v=1}^{m}w_{v}\left(q_{m-v+1}-q_{m-v}\right)}{\left(E_{1}-2\eta \kappa_{1}\tau_{1}\right)\left(E_{1}+2\eta \kappa_{1}\tau_{1}\right)-36\gamma_{1}\gamma_{2}}\\ -\frac{\left(E_{1}+2\eta \kappa_{1}\tau_{1}\right)\sum_{v=1}^{m}w_{v}\left(p_{m-v+1}-p_{m-v}\right)}{\left(E_{1}-2\eta \kappa_{1}\tau_{1}\right)\left(E_{1}+2\eta \kappa_{1}\tau_{1}\right)-36\gamma_{1}\gamma_{2}} \end{array}$$ (39)

$$\begin{array}{c} q_{m+1}=\frac{q_{m}\left(E_{1}\left(E_{1}-2\eta \kappa_{1}\tau_{1}\right)+6\gamma_{2}\left(2\eta \tau_{1}^{2}+2\eta \kappa_{1}^{2}\right)\right)}{\left(E_{1}-2\eta \kappa_{1}\tau_{1}\right)\left(E_{1}+2\eta \kappa_{1}\tau_{1}\right)-36\gamma_{1}\gamma_{2}}\\ +\frac{p_{m}\left(E_{1}-2\eta \kappa_{1}\tau_{1}\right)\left(2\eta \tau_{1}^{2}+2\eta \kappa_{1}^{2}\right)+6\gamma_{2}E_{1})}{\left(E_{1}-2\eta \kappa_{1}\tau_{1}\right)\left(E_{1}+2\eta \kappa_{1}\tau_{1}\right)-36\gamma_{1}\gamma_{2}}-\frac{\left(E_{1}-2\eta \kappa_{1}\tau_{1}\right)\sum_{v=1}^{m}w_{v}\left(q_{m-v+1}-q_{m-v}\right)}{\left(E_{1}-2\eta \kappa_{1}\tau_{1}\right)\left(E_{1}+2\eta \kappa_{1}\tau_{1}\right)-36\gamma_{1}\gamma_{2}}\\ +\frac{6\gamma_{2}\sum_{v=1}^{m}w_{v}\left(p_{m-v+1}-p_{m-v}\right)}{\left(E_{1}-2\eta \kappa_{1}\tau_{1}\right)\left(E_{1}+2\eta \kappa_{1}\tau_{1}\right)-36\gamma_{1}\gamma_{2}} \end{array}$$ (40)

Assuming that Δ*t* is sufficiently small so that *E*_1_ → 0, we get

$$\begin{array}{c} p_{1}\to p_{0},\;q_{1}\to q_{0},\;p_{m+1}\to p_{m}-\sum_{v=1}^{m}w_{v}\left(p_{m-v+1}-p_{m-v}\right),\;q_{m+1}\to q_{m}-\sum_{v=1}^{m}w_{v}\left(q_{m-v+1}-q_{m-v}\right) \end{array}$$ (41)

Using (1) and iterative formulae in the Eq (41) we get |*p*_*m*+1_| ≤ |*p*_0_|, |*q*_*m*+1_| ≤ |*q*_0_|, for *m* = 0, 1, 2, ….

## 5 Analysis and results of examples

Approximate results are revealed to show the perfection of the proposed methodology through *L*_2_(*N*), *L*_∞_(*N*) in this section, which are defined as

$$\begin{array}{c} L_{2}\left(N\right)={\left\|\phi \left(\xi_{r},t\right)-\hat{\phi }\left(\xi_{r},t\right)\right\|}_{2}=\sqrt{h\sum_{r=0}^{N}{\left|\phi \left(\xi_{r},t\right)-\hat{\phi }\left(\xi_{r},t\right)\right|}^{2}}, \end{array}$$

$$\begin{array}{c} L_{\infty }\left(N\right)=\|\phi \left(\xi_{r},t\right)-\hat{\phi }\left(\xi_{r},t\right){\|}_{\infty }=\underset{0\leq r\leq N}{\text{max}}\left|\phi \left(\xi_{r},t\right)-\hat{\phi }\left(\xi_{r},t\right)\right| \end{array}$$

and order of the convergence is calculated using following formula

$$\begin{array}{c} {\text{log}}_{2}\left(\frac{L_{\infty }\left(2h,2\Delta t\right)}{L_{\infty }\left(h,\Delta t\right)}\right). \end{array}$$

Every example is examined by considering *AB*(*γ*) = 1.

**Example 4.1** *Consider the TFSEE for*

$$\begin{array}{c} \dot{\iota }\frac{\partial^{\gamma }G\left(\xi ,t\right)}{\partial t^{\gamma }}+\frac{\partial^{2}G\left(\xi ,t\right)}{\partial \xi^{2}}+{\left|G\left(\xi ,t\right)\right|}^{2}G\left(\xi ,t\right)=U\left(\xi ,t\right),\;\;a\leq \xi \leq b,\;\;t\in \left[t_{0},T\right], \end{array}$$

*with IC and BCs*

$$\begin{array}{c} \left\{ \begin{array}{c} G(\xi ,0)=0,\\ G(0,t)=0,\\ G\left(1,t\right)=t^{2}\left(1+\dot{\iota }\right), \end{array}\right. \end{array}$$ (42)

*and calculation of source function U is*

$$\begin{array}{c} U\left(\xi ,t\right)=-(\frac{2}{1-\gamma }{\left(\xi \right)}^{2}t^{2}E_{\gamma ,3}\left[\frac{-\gamma t^{\gamma }}{1-\gamma }\right]-6\xi t^{2}-\xi^{3}t^{2}\left(\xi^{6}t^{4}+\xi^{4}t^{4}\right))\\ +\dot{\iota }\left(\frac{2}{1-\gamma }{\left(\xi \right)}^{3}t^{2}E_{\gamma ,3}\left[\frac{-\gamma t^{\gamma }}{1-\gamma }\right]+2t^{2}+\xi^{2}t^{2}\left(\xi^{6}t^{4}+\xi^{4}t^{4}\right)\right). \end{array}$$

**Fig 1:**
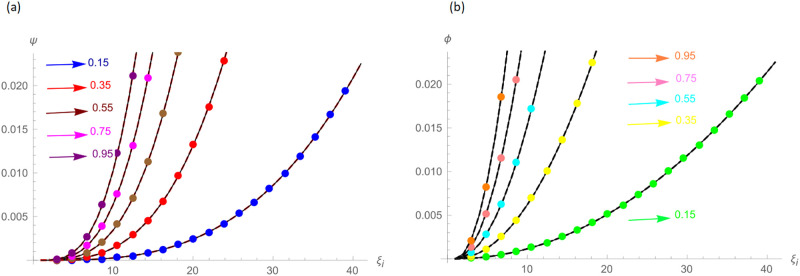
Approximate and analytic solutions with Δ*t* = 0.002 of Example 4.1 at various time levels, (a): Real Part For *N* = 40, *γ* = 0.55, (b): Imaginary Part For *N* = 40, *γ* = 0.55.

**Fig 2:**
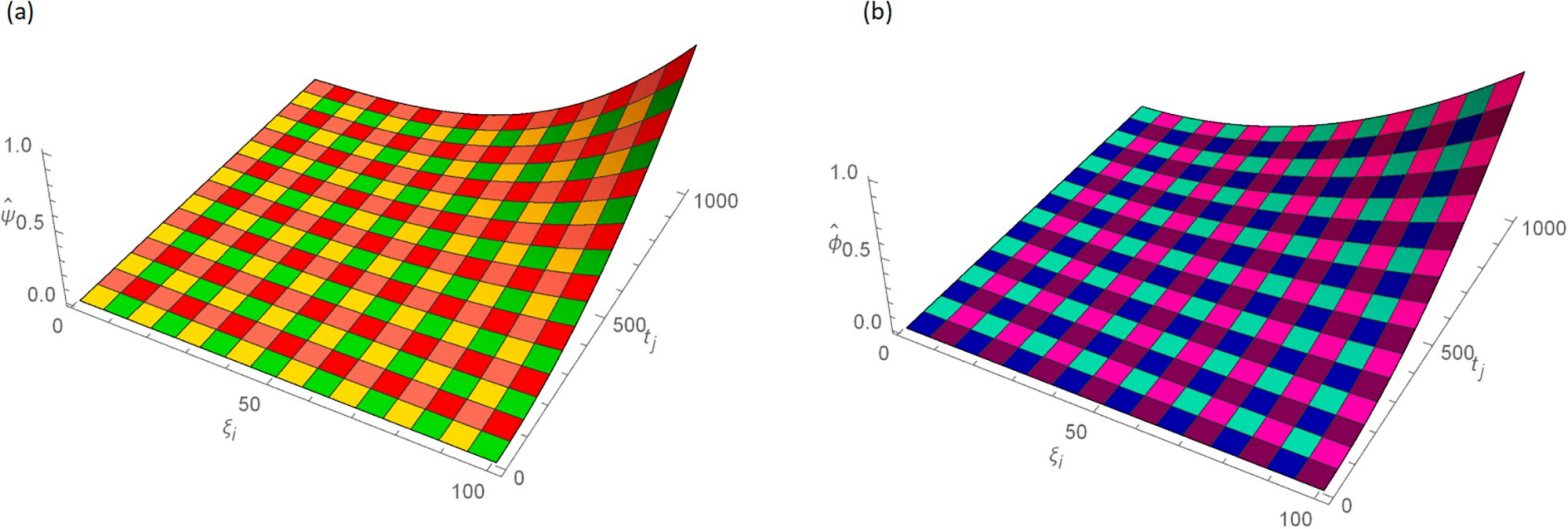
3D analytic solution of real and imaginary parts images with *N* = 100, *t* = 1, *γ* = 0.55, Δ*t* = 0.001 for Example 4.1 when 0 ≤ *ξ* ≤ 1, (a): $\hat{\psi }$, (b): $\hat{\phi }$.

**Fig 3:**
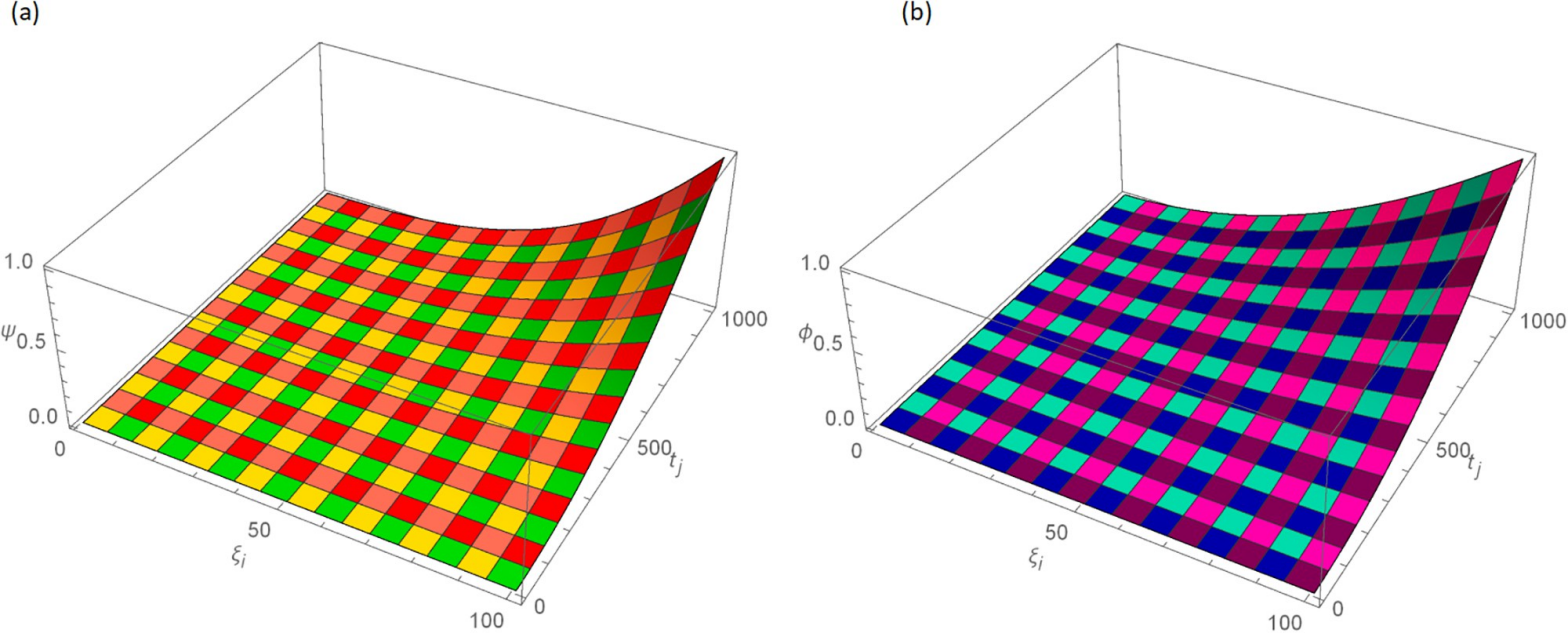
3D approximate solution of real and imaginary parts images with *N* = 100, *t* = 1, *γ* = 0.55, Δ*t* = 0.001 for Example 4.1 when 0 ≤ *ξ* ≤ 1, (a): *ψ*, (b): *ϕ*.

**Fig 4:**
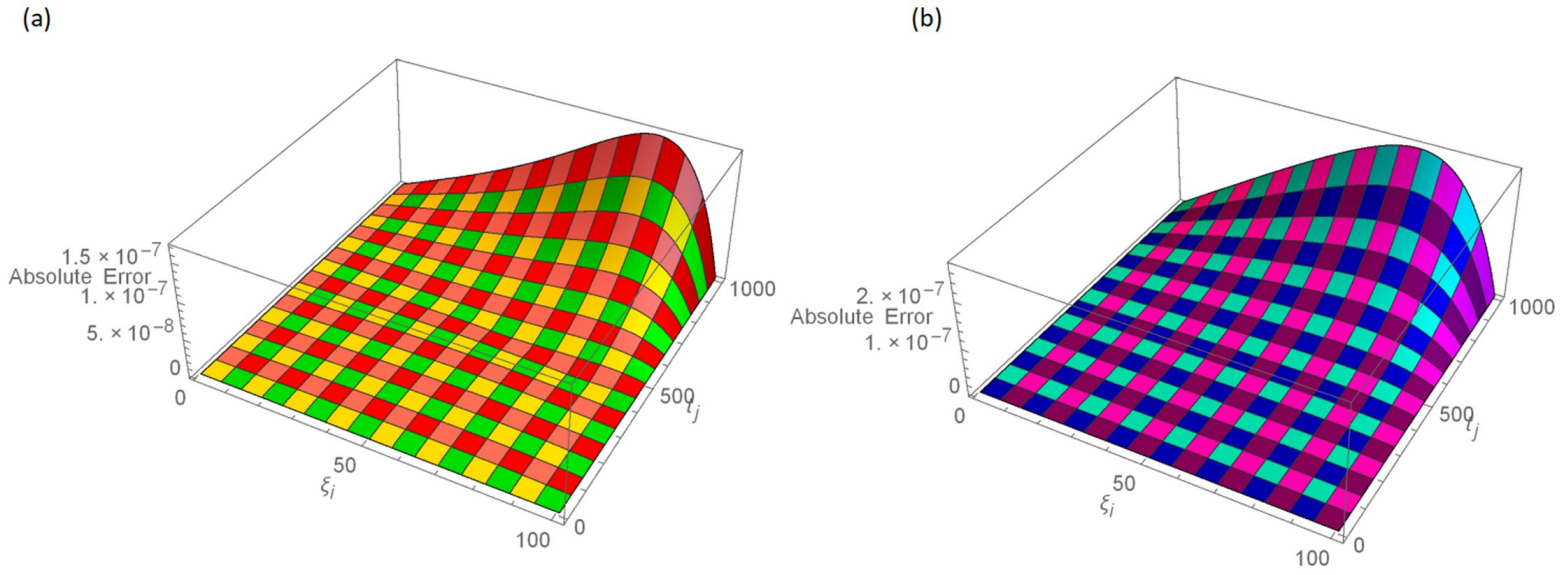
3D error images with *N* = 100, *t* = 1, *γ* = 0.55, Δ*t* = 0.001 for Example 4.1 when 0 ≤ *ξ* ≤ 1, (a): $\hat{\psi }$, (b): $\hat{\phi }$.

**Fig 5:**
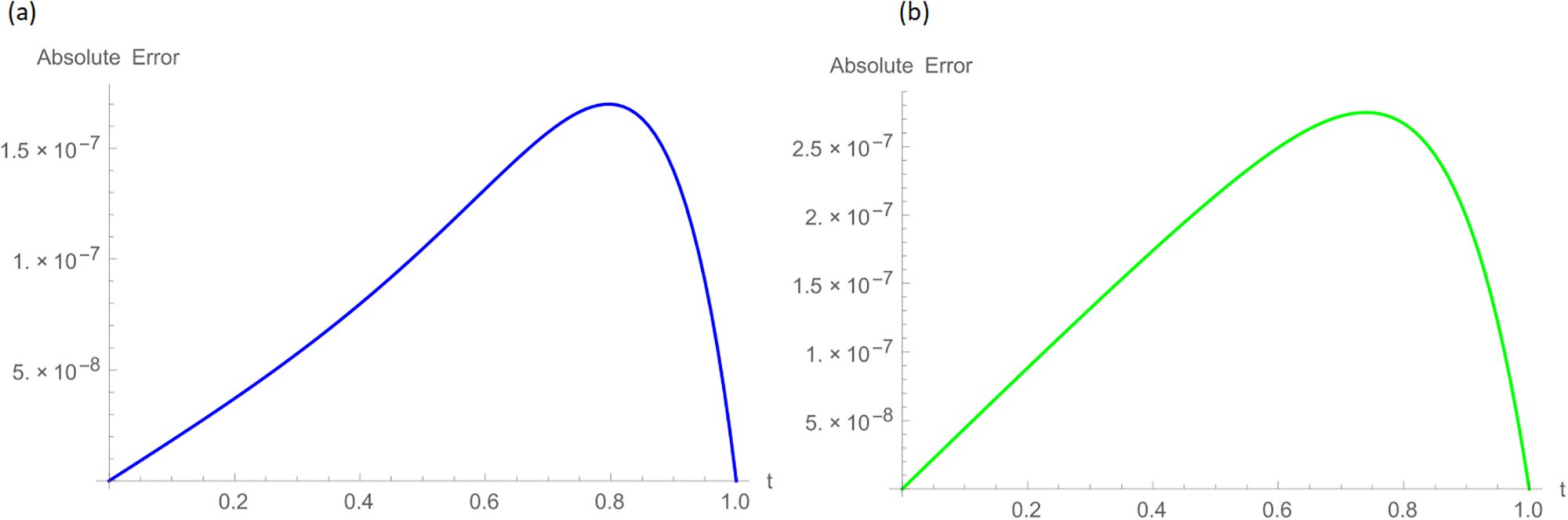
2D error images with *N* = 100, *t* = 1, *γ* = 0.55, Δ*t* = 0.001 for Example 4.1 when 0 ≤ *ξ* ≤ 1, (a): $\hat{\psi }$, (b): $\hat{\phi }$.

The analytical solution is $G\left(\xi ,t\right)=\xi^{3}t^{2}+\dot{\iota }\xi^{2}t^{2}$. Numerical results and error norms for different values of *γ* with *N* = 40, Δ*t* = 0.002, and *t* = 1 for example, 4.1, are presented in Tables 1 and 2. Numerical solution and absolute errors for real and imaginary parts of 4.1, with *γ* = 0.005, *N* = 100, and Δ*t* = 0.0005, are shown in Table 3. For various time levels and *γ* = 0.555, the error norms of real and imaginary parts are shown in Table 4. The order of convergence is shown in Tables 5 and 6. The obtained results of the proposed method and analytic solution have a closed commitment as shown in Fig 1 for different values of time *t* with Δ*t* = 0.002. Figs 2 and 3 for *N* = 100, Δ*t* = 0.001, *γ* = 0.55, *t* = 1, and *ξ* ∈ [0, 1] show the 3D plot of analytic solutions and numerical solutions, respectively. The 2D error plots are presented in Figs 4 and 5 at *t* = 1, respectively.

**Table 1:** Comparison of numerical solution and analytic solution of real part of Example 4.1 with error norms for different values of *γ*, *N* = 40, $\Delta t=\frac{1}{500}$ and 0 ≤ *ξ* ≤ 1.

*ξ*	$\hat{\psi }$	*ψ*			
		*γ* = 0.275	*γ* = 0.475	*γ* = 0.675	*γ* = 0.875
0.1	0.00100	0.00099	0.00099	0.00099	0.00100
0.2	0.00800	0.00799	0.00799	0.00799	0.00800
0.3	0.02700	0.02699	0.02699	0.02699	0.02699
0.4	0.06400	0.06399	0.06399	0.06399	0.06399
0.5	0.12500	0.12499	0.12499	0.12499	0.12499
0.6	0.21600	0.21599	0.21599	0.21599	0.21599
0.7	0.34300	0.34299	0.34299	0.34299	0.34299
0.8	0.51200	0.51199	0.51199	0.51199	0.51199
0.9	0.72900	0.72899	0.72899	0.72899	0.72899
**L**_∞_	…	7.31412 × 10^−7^	6.99348 × 10^−7^	6.32884 × 10^−7^	4.49627 × 10^−7^
**L**_**2**_	…	4.70535 × 10^−7^	4.42871 × 10^−7^	3.85365 × 10^−7^	2.28939 × 10^−7^

**Table 2:** Comparison of numerical solutions and analytic solutions of the imaginary part of Example 4.1 with error norms for different values of *γ*, *N* = 40, $\Delta t=\frac{1}{500}$ and 0 ≤ *ξ* ≤ 1.

*ξ*	$\hat{\phi }$	*ϕ*			
		*γ* = 0.275	*γ* = 0.475	*γ* = 0.675	*γ* = 0.875
0.1	0.00100	0.00999	0.00999	0.00999	0.00999
0.2	0.04000	0.03999	0.03999	0.03999	0.03999
0.3	0.09000	0.08999	0.08999	0.08999	0.08999
0.4	0.16000	0.15999	0.15999	0.15999	0.15999
0.5	0.25000	0.24999	0.24999	0.24999	0.24999
0.6	0.36000	0.35999	0.35999	0.35999	0.35999
0.7	0.49000	0.48999	0.48999	0.48999	0.48999
0.8	0.64000	0.63999	0.63999	0.63999	0.63999
0.9	0.81000	0.80999	0.80999	0.80999	0.80999
**L**_∞_	…	1.06884 × 10^−6^	1.08780 × 10^−6^	1.12622 × 10^−6^	1.24737 × 10^−6^
**L**_**2**_	…	7.2692 × 10^−7^	7.41115 × 10^−7^	7.69361 × 10^−7^	8.54957 × 10^−7^

**Table 3:** Errors with Δ*t* = 0.0005 of Example 4.1 at *t* = 1.

*ξ*	*γ* = 0.005, *N* = 100			*γ* = 0.005, *N* = 100		
	$\hat{\psi }$	*ψ*	Absolute Error	$\hat{\phi }$	*ϕ*	Absolute Error
0.1	0.00100	0.00099	5.90486 × 10^−9^	0.01000	0.00999	1.05354 × 10^−8^
0.2	0.00800	0.00799	1.19214 × 10^−8^	0.04000	0.03999	2.10060 × 10^−8^
0.3	0.02700	0.02699	1.81465 × 10^−8^	0.09000	0.08999	3.13523 × 10^−8^
0.4	0.06400	0.06399	2.46704 × 10^−8^	0.16000	0.15999	4.14749 × 10^−8^
0.5	0.12500	0.12499	3.15166 × 10^−8^	0.25000	0.24999	5.11389 × 10^−8^
0.6	0.21600	0.21599	3.84585 × 10^−8^	0.36000	0.35999	5.96985 × 10^−8^
0.7	0.34300	0.34299	4.45078 × 10^−8^	0.49000	0.48999	6.54707 × 10^−8^
0.8	0.51200	0.51199	4.66924 × 10^−8^	0.64000	0.63999	6.44796 × 10^−8^
0.9	0.72900	0.72899	3.74825 × 10^−8^	0.81000	0.80999	4.82037 × 10^−8^

**Table 4:** *L*_∞_(*N*) and *L*_2_(*N*) for different values of *γ* = 0.555 with $\Delta t=\frac{1}{2000}$, *N* = 100 and 0 ≤ *ξ* ≤ 1 of 4.1.

t	L_∞_(N)		L_2_(N)	
	*ψ*	*ϕ*	*ψ*	*ϕ*
0.1	9.70942 × 10^−11^	6.62294 × 10^−11^	6.83677 × 10^−11^	4.66045 × 10^−11^
0.5	6.49999 × 10^−10^	2.08147 × 10^−9^	3.16909 × 10^−10^	1.42083 × 10^−9^
0.9	3.03875 × 10^−8^	5.00404 × 10^−8^	1.89336 × 10^−8^	3.40952 × 10^−8^

**Table 5:** Order of the convergence with *N* = 100 and *γ* = 0.555 for different values of $\Delta t=\frac{1}{m}$ of 4.1.

m	*ψ*		*ϕ*	
	L_∞_(N)	Order of convergence	L_∞_(N)	Order of convergence
4	5.38124 × 10^−3^	…	9.00228 × 10^−3^	…
8	1.91633 × 10^−3^	1.48959	3.14738 × 10^−3^	1.51614
16	5.67232 × 10^−4^	1.75633	9.25062 × 10^−4^	1.76653
32	1.53729 × 10^−4^	1.88355	2.49905 × 10^−4^	1.88817
64	3.99607 × 10^−5^	1.94374	6.48693 × 10^−5^	1.94577

**Table 6:** Order of the convergence with *γ* = 0.555 for different values of $\Delta t=\frac{1}{m}$ and $h=\frac{1}{n}$ of 4.1.

m = n	*ψ*		*ϕ*	
	L_∞_(N)	Order of convergence	L_∞_(N)	Order of convergence
10	1.40857 × 10^−3^	…	2.24281 × 10^−3^	…
20	3.81521 × 10^−4^	1.88439	6.17968 × 10^−4^	1.85970
40	1.00319 × 10^−4^	1.92716	1.62689 × 10^−4^	1.92541
80	2.57817 × 10^−5^	1.96018	4.18367 × 10^−5^	1.95928
160	6.53823 × 10^−6^	1.97938	1.06081 × 10^−5^	1.97961

**Example 4.2** *Consider the TFSE*

$$\begin{array}{c} \dot{\iota }\frac{\partial^{\gamma }G\left(\xi ,t\right)}{\partial t^{\gamma }}+\frac{\partial^{2}G\left(\xi ,t\right)}{\partial \xi^{2}}+{\left|G\left(\xi ,t\right)\right|}^{2}G\left(\xi ,t\right)=U\left(\xi ,t\right),\;\;a\leq \xi \leq b,\;\;t\in \left[t_{0},T\right], \end{array}$$

*with IC and BCs*

$$\begin{array}{c} \left\{ \begin{array}{c} G(\xi ,0)=0,\\ G(0,t)=0,\\ G\left(1,t\right)=\dot{\iota }t^{2}\xi^{2}, \end{array}\right. \end{array}$$ (43)

*and calculation of source functionU is*

$$\begin{array}{c} U\left(\xi ,t\right)=-(\frac{2}{1-\gamma }{\left(\xi \right)}^{2}t^{2}E_{\gamma ,3}\left[\frac{-\gamma t^{\gamma }}{1-\gamma }\right]+2t^{2}-\left(\xi -\xi^{2}\right)t^{2}\left({\left(\xi -\xi^{2}\right)}^{2}t^{4}+\xi^{4}t^{4}\right))\\ +\dot{\iota }\left(\frac{2}{1-\gamma }\left(\xi -\xi^{2}\right)t^{2}E_{\gamma ,3}\left[\frac{-\gamma t^{\gamma }}{1-\gamma }\right]+2t^{2}+\xi^{2}t^{2}\left({\left(\xi -\xi^{2}\right)}^{2}t^{4}+\xi^{4}t^{4}\right)\right). \end{array}$$

**Fig 6:**
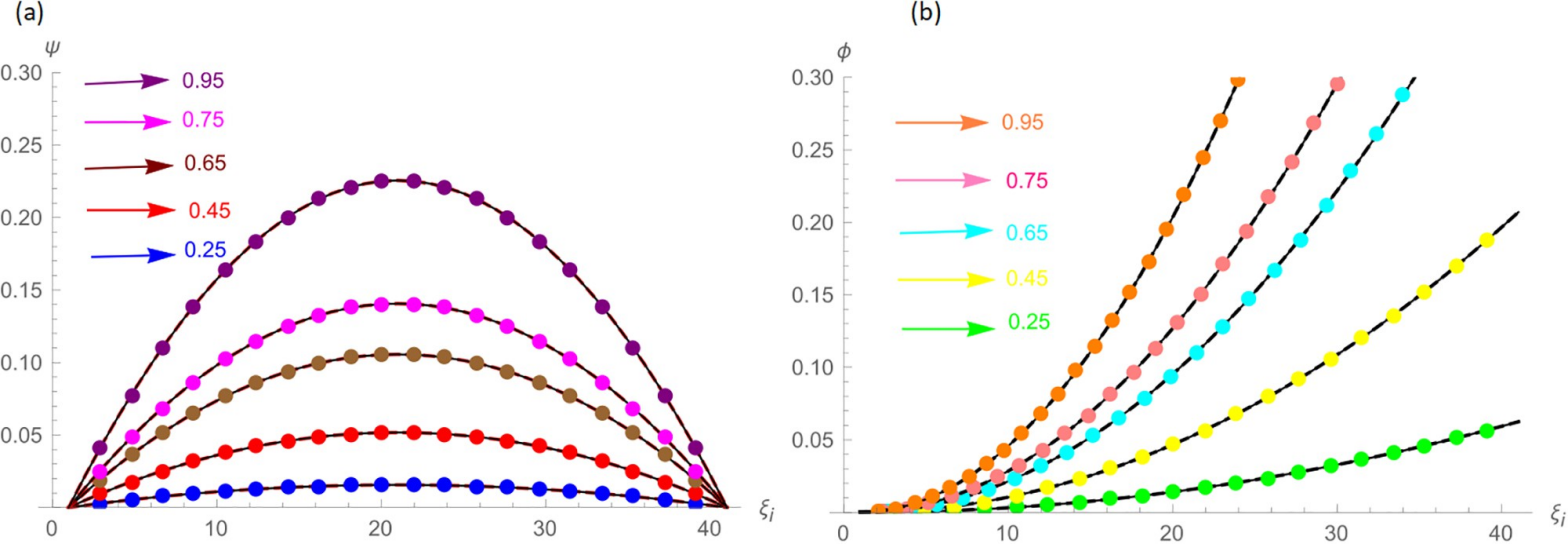
At different time levels the approximate and analytic solutions for Example 4.2, (a): Real Part *N* = 40, *γ* = 0.15 and Δ*t* = 0.002, (b): Imaginary Part *N* = 40, *γ* = 0.15 and Δ*t* = 0.002.

**Fig 7:**
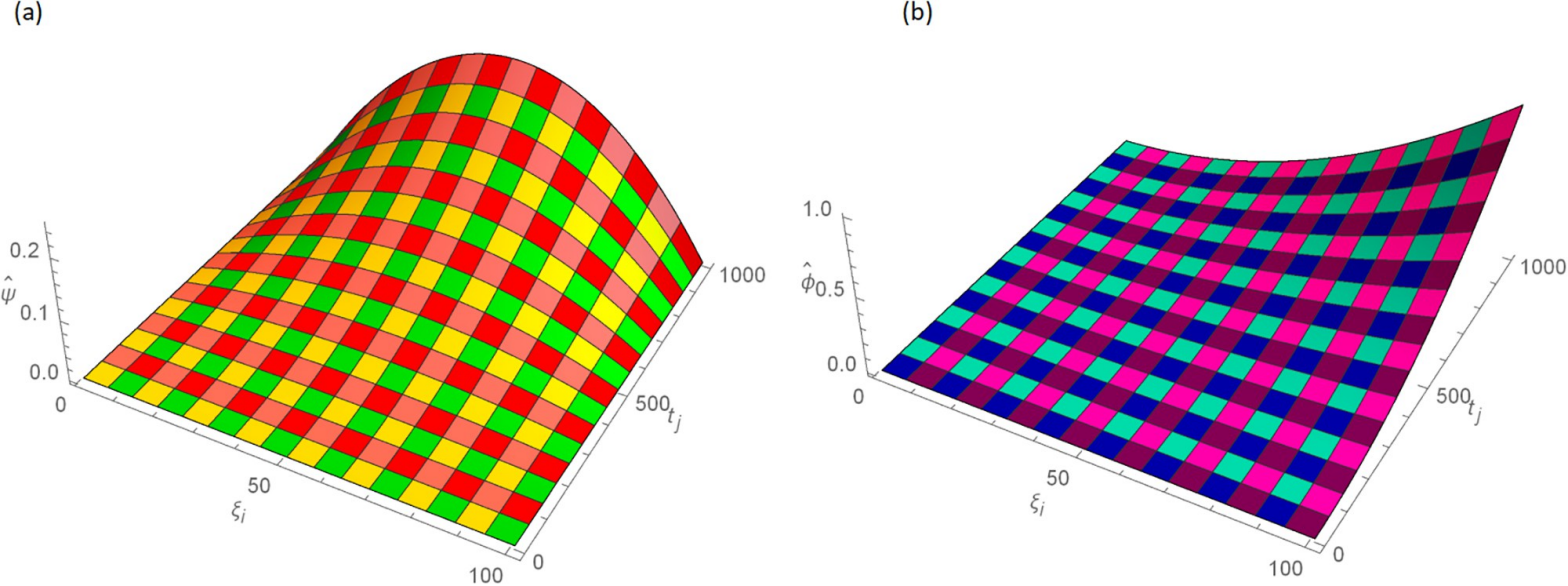
For Example 4.2 the 3D analytic solutions images when *N* = 100, *t* = 1, *γ* = 0.55, $\Delta t=\frac{1}{1000}$ and 0 ≤ *ξ* ≤ 1, (a): $\hat{\psi }$, (b): $\hat{\phi }$.

**Fig 8:**
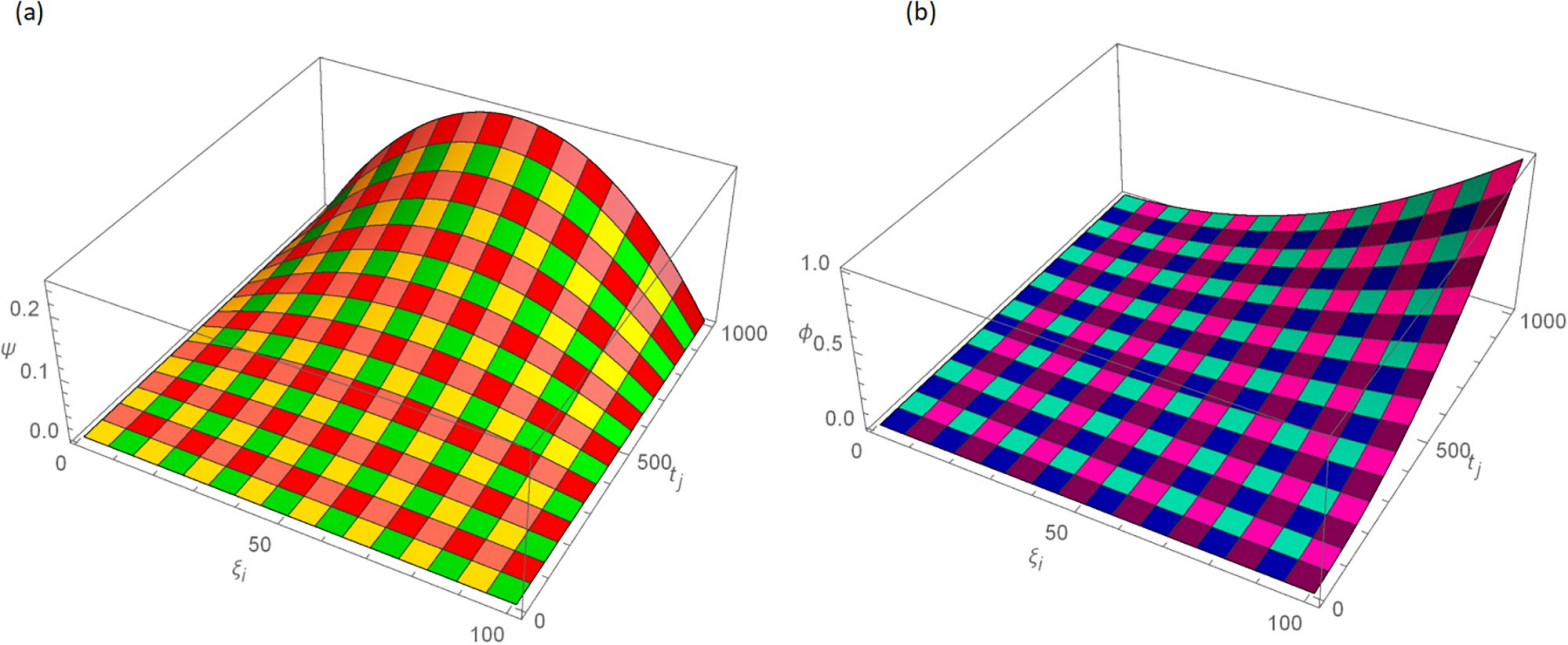
For Example 4.2 the 3D approximate solution images when *N* = 100, *t* = 1, *γ* = 0.55, $\Delta t=\frac{1}{1000}$ and 0 ≤ *ξ* ≤ 1, (a): *ψ*, (b): *ϕ*.

**Fig 9:**
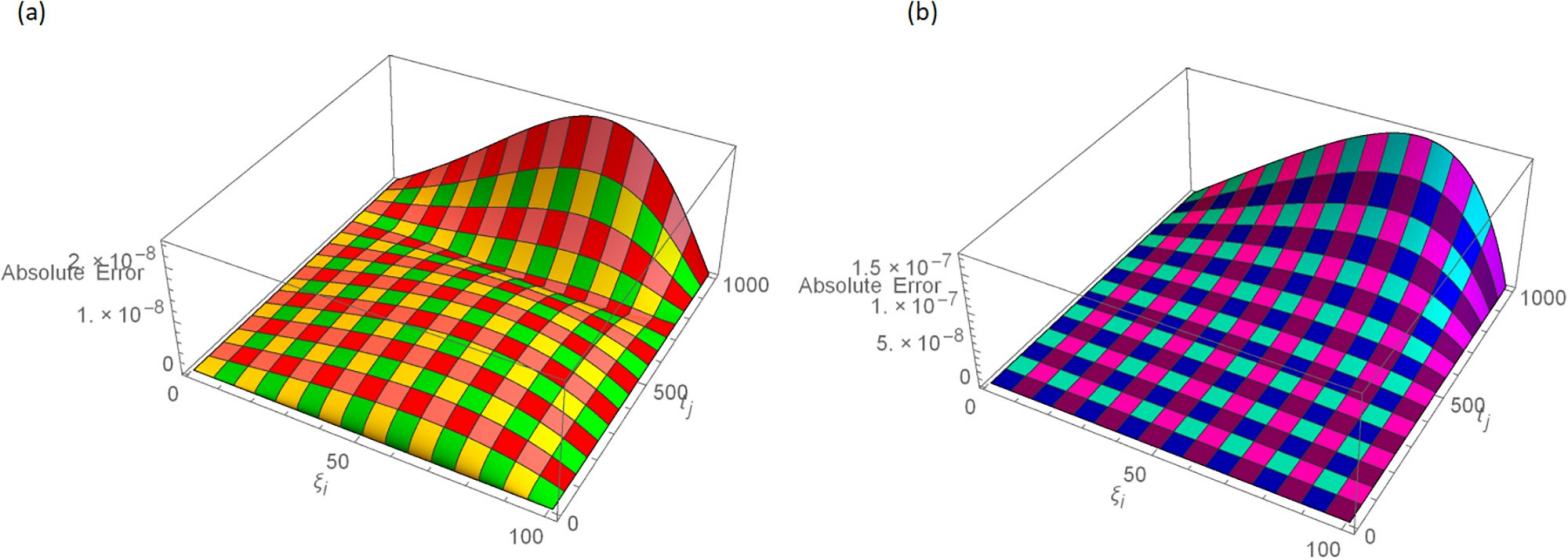
3D error graphs with *N* = 100, *t* = 1, *γ* = 0.55, $\Delta t=\frac{1}{1000}$ for Example 4.2 when 0 ≤ *ξ* ≤ 1, (a): $\hat{\psi }$, (b): $\hat{\phi }$.

**Fig 10:**
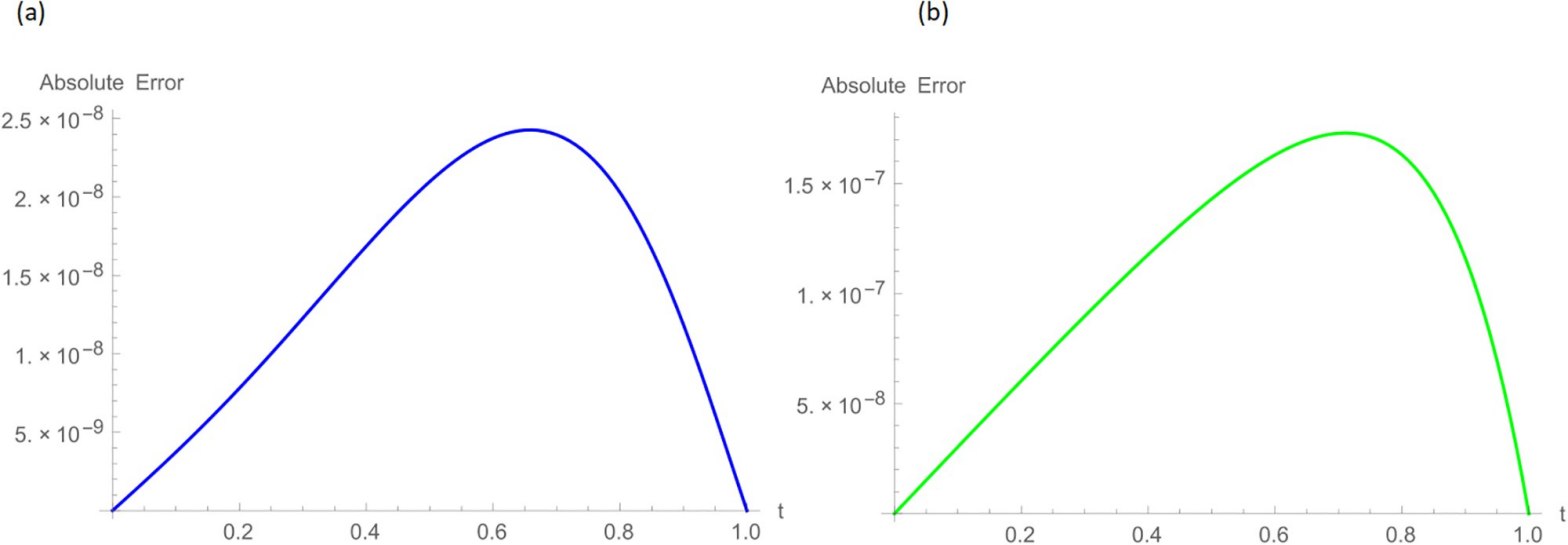
2D error graphs with *N* = 100, *t* = 1, *γ* = 0.55, $\Delta t=\frac{1}{1000}$ for Example 4.2 when 0 ≤ *ξ* ≤ 1, (a): $\hat{\psi }$, (b): $\hat{\phi }$.

The $G\left(\xi ,t\right)=t^{2}\left(\xi -\xi^{2}\right)+\dot{\iota }\xi^{2}t^{2}$ is the analytic solution. Numerical results and error norms of real and imaginary parts for different values of *γ* with *N* = 40, Δ*t* = 0.002 and *t* = 1, for example 4.2, are presented in Tables 7 and 8. Numerical solution and absolute errors for real and imaginary parts of 4.2 with *γ* = 0.005, *N* = 100, and Δ*t* = 0.0005 are shown in Table 9. For various time levels and *γ* = 0.555, the error norms of real and imaginary parts are shown in Table 10. Table 11 presents a comparison of [27] and proposed method for Example 4.2. The order of convergence is shown in Tables 12 and 13. Fig 6 highlights the performance of analytic solutions and numerical outcomes at different temporal directions. 3D plots in Figs 7 and 8 of the exact and computational solutions are presented, respectively. The 3D and 2D error graphs are shown in Figs 9 and 10, respectively.

**Table 7:** Comparison of numerical solution and analytic solution of real part of Example 4.2 with error norms for different values of *γ*, *N* = 40, $\Delta t=\frac{1}{500}$ and 0 ≤ *ξ* ≤ 1.

*ξ*	$\hat{\psi }$	*ψ*			
		*γ* = 0.12	*γ* = 0.42	*γ* = 0.72	*γ* = 0.92
0.1	0.09000	0.08999	0.08999	0.09000	0.09000
0.2	0.16000	0.15999	0.15999	0.16000	0.16000
0.3	0.21000	0.20999	0.20999	0.21000	0.21000
0.4	0.24000	0.23999	0.23999	0.23999	0.24000
0.5	0.25000	0.24999	0.24999	0.24999	0.25000
0.6	0.24000	0.23999	0.23999	0.23999	0.24000
0.7	0.21000	0.20999	0.20999	0.20999	0.21000
0.8	0.16000	0.15999	0.15999	0.15999	0.16000
0.9	0.09000	0.08999	0.08999	0.08999	0.09000
**L**_∞_	…	1.60668 × 10^−7^	1.26563 × 10^−7^	2.50173 × 10^−8^	3.11053 × 10^−7^
**L**_**2**_	…	1.10769 × 10^−7^	8.6238 × 10^−8^	1.36147 × 10^−8^	2.23728 × 10^−7^

**Table 8:** Comparison of numerical solutions and analytic solutions of imaginary part *ϕ* of Example 4.2 with error norms for different values of *γ*, *N* = 40, $\Delta t=\frac{1}{500}$ and 0 ≤ *ξ* ≤ 1.

*ξ*	$\hat{\phi }$	*ϕ*			
		*γ* = 0.12	*γ* = 0.42	*γ* = 0.72	*γ* = 0.92
0.1	0.01000	0.00999	0.00999	0.00999	0.00999
0.2	0.04000	0.03999	0.03999	0.03999	0.03999
0.3	0.09000	0.08999	0.08999	0.08999	0.08999
0.4	0.16000	0.15999	0.15999	0.15999	0.15999
0.5	0.25000	0.24999	0.24999	0.24999	0.24999
0.6	0.36000	0.35999	0.35999	0.35999	0.35999
0.7	0.49000	0.48999	0.48999	0.48999	0.48999
0.8	0.64000	0.63999	0.63999	0.63999	0.63999
0.9	0.81000	0.80999	0.80999	0.80999	0.80999
**L**_∞_	…	6.73361 × 10^−7^	6.82665 × 10^−7^	7.11759 × 10^−7^	8.45041 × 10^−7^
**L**_**2**_	…	4.64116 × 10^−7^	4.72162 × 10^−7^	4.96083 × 10^−7^	6.03905 × 10^−7^

**Table 9:** Errors with Δ*t* = 0.0005 of Example 4.2 at *t* = 1.

*ξ*	*γ* = 0.005, *N* = 100			*γ* = 0.005, *N* = 100		
	$\hat{\psi }$	*ψ*	Absolute Error	$\hat{\phi }$	*ϕ*	Absolute Error
0.1	0.09000	0.08999	1.98729 × 10^−9^	0.01000	0.00999	7.13544 × 10^−9^
0.2	0.16000	0.15999	4.00700 × 10^−9^	0.04000	0.03999	1.42440 × 10^−8^
0.3	0.21000	0.20999	6.02548 × 10^−9^	0.09000	0.08999	2.12680 × 10^−8^
0.4	0.24000	0.23999	7.90477 × 10^−9^	0.16000	0.15999	2.80605 × 10^−8^
0.5	0.25000	0.24999	9.42228 × 10^−9^	0.25000	0.24999	3.43063 × 10^−8^
0.6	0.24000	0.23999	1.02725 × 10^−8^	0.36000	0.35999	3.93762 × 10^−8^
0.7	0.21000	0.20999	1.00666 × 10^−8^	0.49000	0.48999	4.20382 × 10^−8^
0.8	0.16000	0.15999	8.37816 × 10^−9^	0.64000	0.63999	3.99158 × 10^−8^
0.9	0.09000	0.08999	4.92427 × 10^−9^	0.81000	0.80999	2.85566 × 10^−8^

**Table 10:** *L*_∞_(*N*) and *L*_2_(*N*) for different values of *γ* = 0.555 with $\Delta t=\frac{1}{2000}$, *N* = 100 and 0 ≤ *ξ* ≤ 1 of 4.2.

t	L_∞_(N)	*ϕ*	L_2_(N)	
	*ψ*		*ψ*	*ϕ*
0.1	1.18086 × 10^−11^	8.36931 × 10^−12^	5.30896 × 10^−12^	3.76617 × 10^−12^
0.5	4.05408 × 10^−10^	2.89885 × 10^−10^	8.42777 × 10^−10^	6.08940 × 10^−10^
0.9	2.39725 × 10^−9^	1.57261 × 10^−9^	2.28225 × 10^−8^	1.58554 × 10^−8^

**Table 11:** Comparison of *L*_∞_ of Example 4.2 for various values of $\Delta t=\frac{1}{m}$ with *γ* = 0.1, *N* = 40 and 0 ≤ *ξ* ≤ 1.

m	Ref [27]		Proposed Method	
	*ψ*	*ϕ*	*ψ*	*ϕ*
16	1.69582 × 10^−2^	1.64917 × 10^−2^	1.36589 × 10^−4^	5.67258 × 10^−4^
32	8.50622 × 10^−3^	8.22627 × 10^−3^	3.68956 × 10^−5^	1.53204 × 10^−4^
64	4.26873 × 10^−3^	4.11967 × 10^−3^	9.57495 × 10^−6^	3.97713 × 10^−5^
128	2.15140 × 10^−3^	2.07201 × 10^−3^	2.43740 × 10^−6^	1.01293 × 10^−5^
256	1.09340 × 10^−3^	1.05034 × 10^−3^	6.14658 × 10^−7^	2.55585 × 10^−6^
512	5.64571 × 10^−4^	5.40111 × 10^−4^	1.54287 × 10^−7^	6.41927 × 10^−7^

**Table 12:** Order of the Convergence with *N* = 120 and *γ* = 0.005 for different values of $\Delta t=\frac{1}{m}$ of Example 4.2.

m	*ψ*		*ϕ*	
	L_∞_(N)	Order of convergence	L_∞_(N)	Order of convergence
4	1.35359 × 10^−3^	…	5.52518 × 10^−3^	…
8	4.73012 × 10^−4^	1.51685	1.92789 × 10^−3^	1.51899
16	1.38923 × 10^−4^	1.76759	5.65836 × 10^−4^	1.76857
32	3.75289 × 10^−5^	1.88821	1.52821 × 10^−4^	1.88854
64	9.74276 × 10^−6^	1.94560	3.96709 × 10^−5^	1.94569

**Table 13:** Order of the convergence with *γ* = 0.005 for different values of $\Delta t=\frac{1}{m}$ and $h=\frac{1}{n}$ of Example 4.2.

m = n	*ψ*		*ϕ*	
	L_∞_(N)	Order of convergence	L_∞_(N)	Order of convergence
10	3.14217 × 10^−4^	…	1.35906 × 10^−3^	…
20	9.11356 × 10^−5^	1.78567	3.76072 × 10^−4^	1.85353
40	2.43442 × 10^−5^	1.90444	9.94374 × 10^−5^	1.91915
80	6.28067 × 10^−6^	1.95459	2.55841 × 10^−5^	1.95854
160	1.59398 × 10^−6^	1.97828	6.48911 × 10^−6^	1.97915

**Example 4.3** *Consider the TFSE as* [27]

$$\begin{array}{c} \dot{\iota }\frac{\partial^{\gamma }G\left(\xi ,t\right)}{\partial t^{\gamma }}+\frac{\partial^{2}G\left(\xi ,t\right)}{\partial \xi^{2}}+{\left|G\left(\xi ,t\right)\right|}^{2}G\left(\xi ,t\right)=U\left(\xi ,t\right),\;\;a\leq \xi \leq b,\;\;t\in \left[t_{0},T\right], \end{array}$$

*with IC and BCs*

$$\begin{array}{c} \left\{ \begin{array}{c} G(\xi ,0)=0,\\ G\left(0,t\right)=\dot{\iota }t,\\ G\left(1,t\right)=t^{3}+\dot{\iota }te^{-1}, \end{array}\right. \end{array}$$ (44)

*and calculation of source function U is*

$$\begin{array}{c} U\left(\xi ,t\right)=-(\frac{1}{1-\gamma }te^{-\xi^{2}}E_{\gamma ,2}\left[\frac{-\gamma t^{\gamma }}{1-\gamma }\right]-6\xi^{3}t^{3}-\xi^{3}t^{3}\left(t^{6}\xi^{6}+e^{-2\xi^{2}}t^{2}\right))\\ +\dot{\iota }\left(\frac{6}{1-\gamma }\xi^{3}t^{3}E_{\gamma ,3}\left[\frac{-\gamma t^{\gamma }}{1-\gamma }\right]+t\left(-2+4\xi^{2}\right)e^{-\xi^{2}}+te^{-\xi^{2}}\left(t^{6}\xi^{6}+e^{-2\xi^{2}}t^{2}\right)\right). \end{array}$$

**Fig 11:**
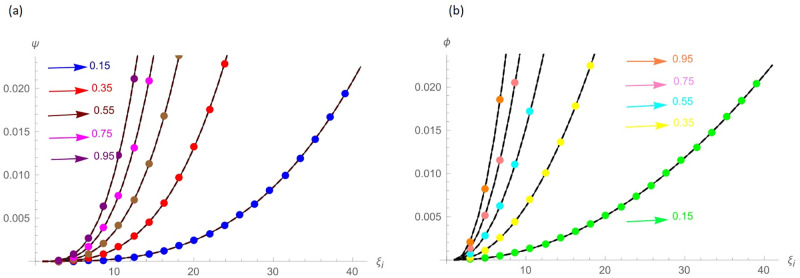
For Example 4.3, the analytic and numerical solution at various temporal directions, (a): Real Part *N* = 100, *γ* = 0.25 and Δ*t* = 0.001, (b): Imaginary Part *N* = 100, *γ* = 0.25 and Δ*t* = 0.001.

**Fig 12:**
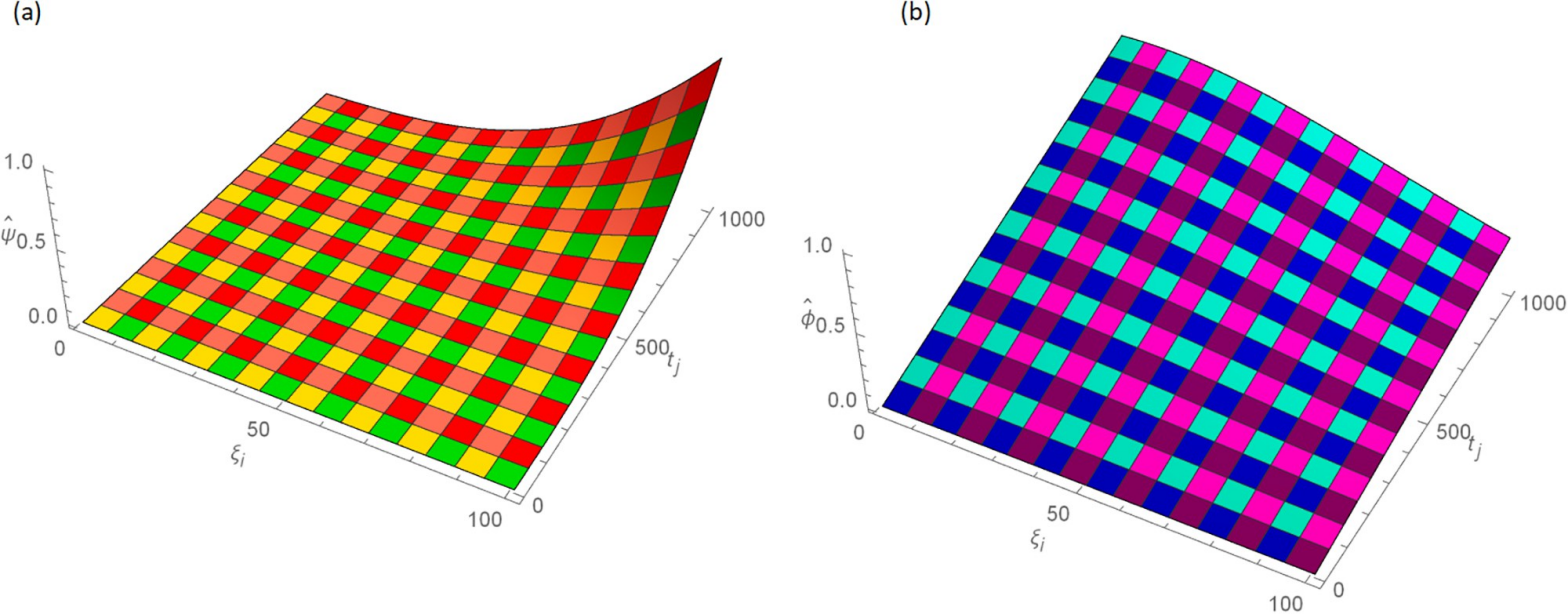
3D analytic solution images with *N* = 100, *t* = 1, *γ* = 0.25, and Δ*t* = 0.001 for Example 4.3 when 0 ≤ *ξ* ≤ 1, (a): $\hat{\psi }$, (b): $\hat{\phi }$.

**Fig 13:**
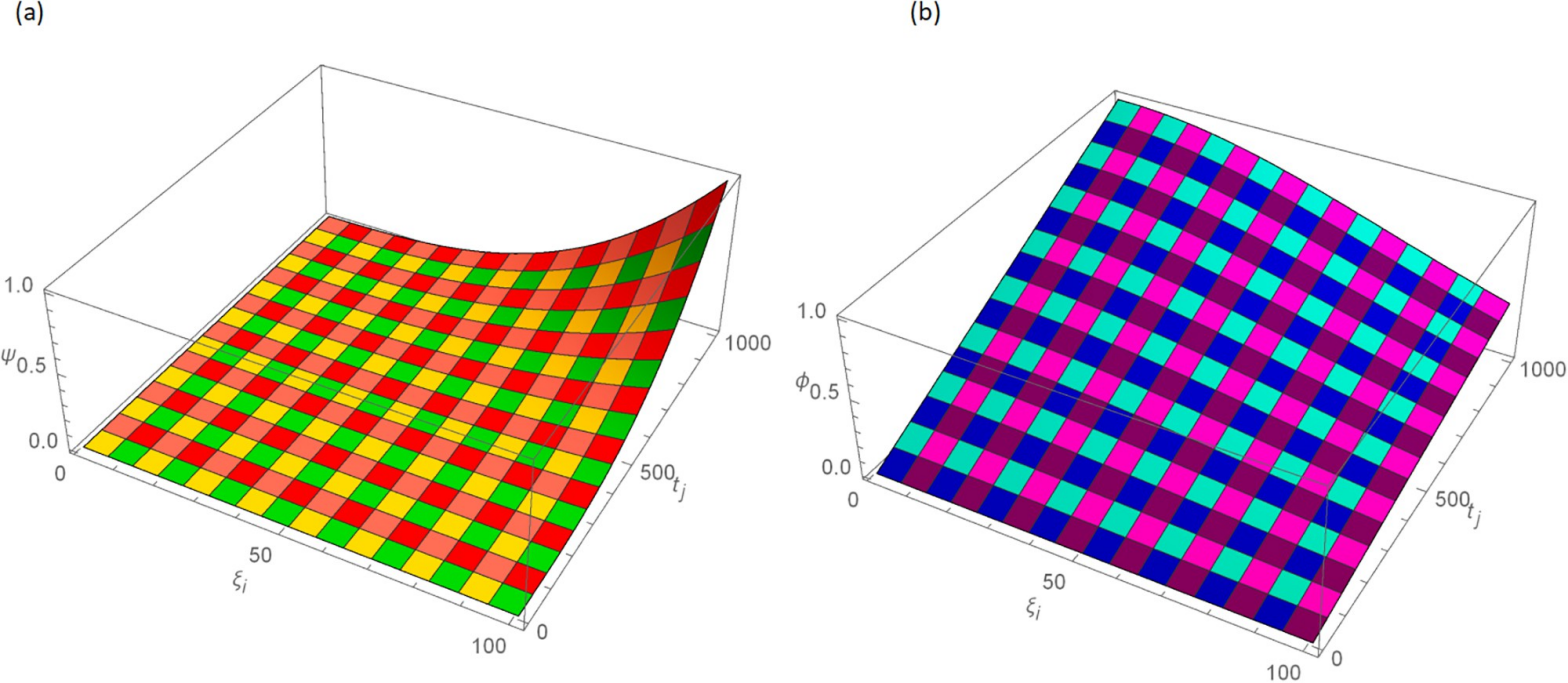
3D numerical solution images with *N* = 100, *t* = 1, *γ* = 0.25, and Δ*t* = 0.001 for Example 4.3 when 0 ≤ *ξ* ≤ 1, (a): *ψ*, (b): *ϕ*.

**Fig 14:**
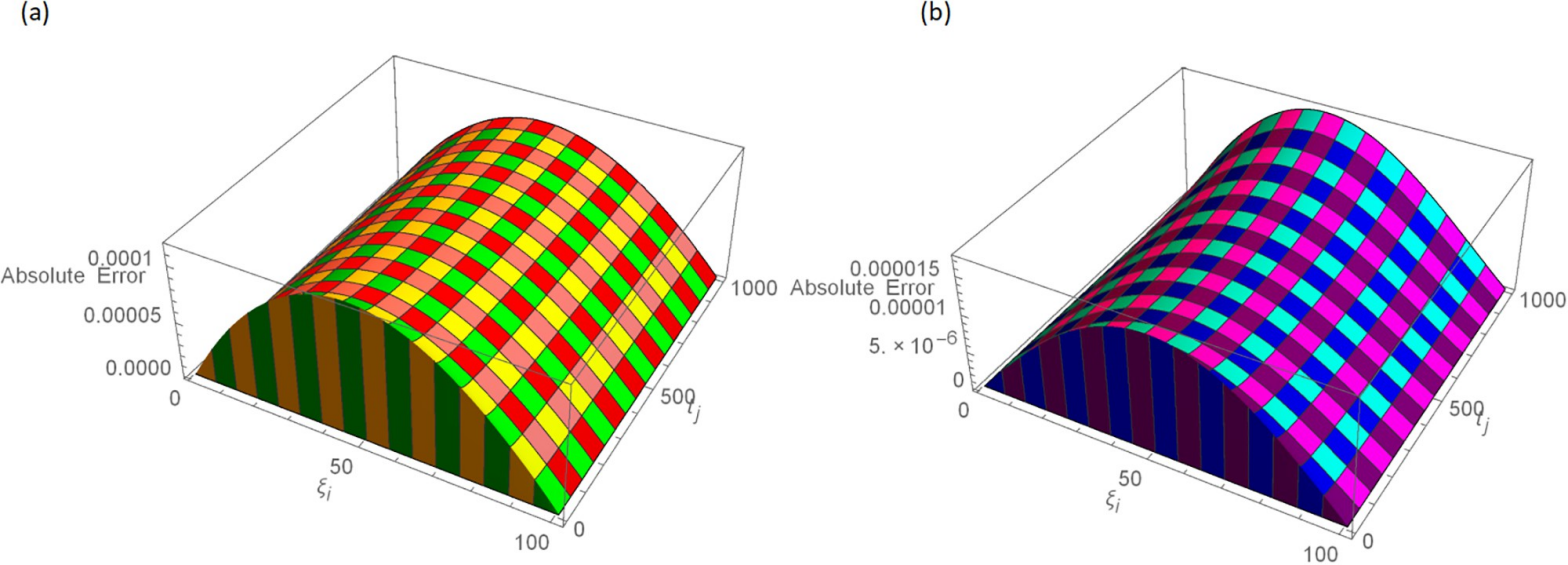
3D error images with *N* = 100, *t* = 1, *γ* = 0.25, and Δ*t* = 0.001 for Example 4.3 when 0 ≤ *ξ* ≤ 1, (a): $\hat{\psi }$, (b): $\hat{\phi }$.

**Fig 15:**
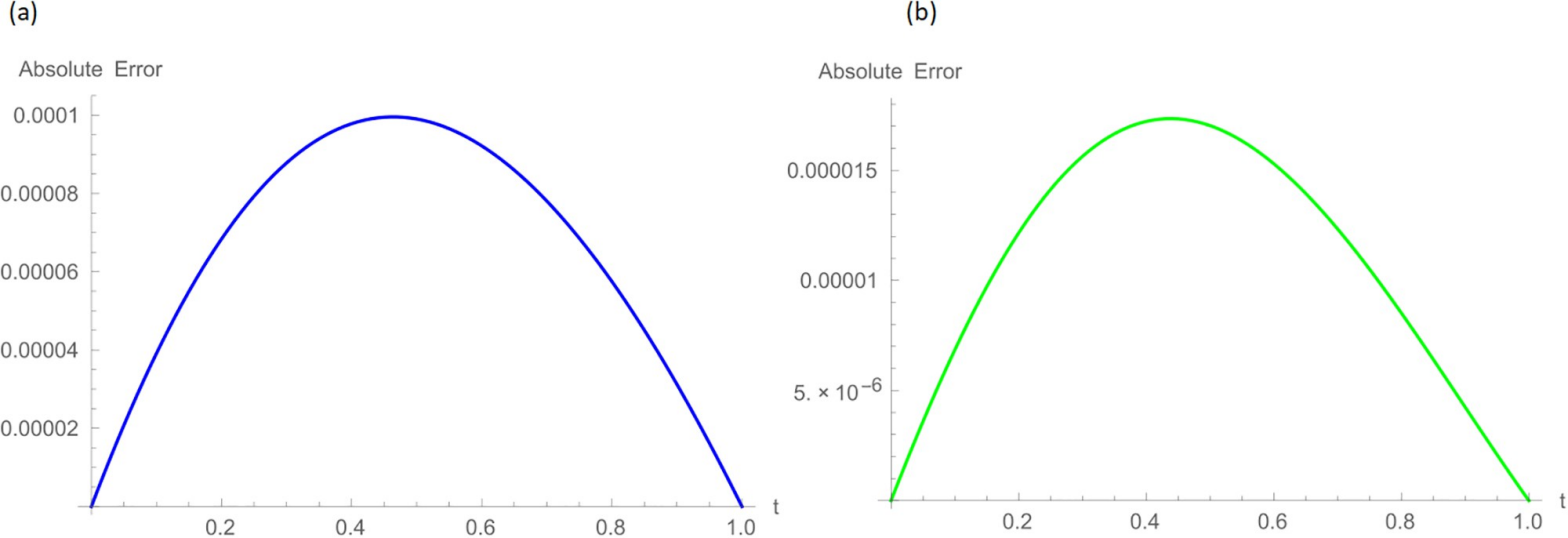
2D error images with *N* = 100, *t* = 1, *γ* = 0.25, and Δ*t* = 0.001 for Example 4.3 when 0 ≤ *ξ* ≤ 1, (a): $\hat{\psi }$, (b): $\hat{\phi }$.

The $G\left(\xi ,t\right)=t^{3}\xi^{3}+\dot{\iota }te^{-\xi^{2}}$ is an analytic solution. Numerical results and error norms of real and imaginary parts for different values of *γ* with *N* = 150, Δ*t* = 0.001 and *t* = 1, for example 4.3, are presented in Tables 14 and 15. Numerical solution and absolute errors for real and imaginary parts of 4.3 with *γ* = 0.875, *N* = 200, and Δ*t* = 0.0005 are shown in Table 16. For various time levels and *γ* = 0.8755, the error norms of real and imaginary parts are shown in Table 17. Fig 11 provides a description of exact values and computational outcomes at different time levels. The 3D precision of the existing approach is demonstrated by graphs of analytical solutions and numerical results in Figs 12–15 demonstrate the 3D and 2D error descriptions, proving the method’s efficiency.

**Table 14:** Comparison of numerical solution and analytic solution of real part with error norms for different values of *γ*, *N* = 150, $\Delta t=\frac{1}{1000}$ and 0 ≤ *ξ* ≤ 1 for Example 4.3.

*ξ*	$\hat{\psi }$	*ψ*			
		*γ* = 0.8	*γ* = 0.85	*γ* = 0.9	*γ* = 0.95
0.1	0.00100	0.00098	0.00098	0.00099	0.00099
0.2	0.00800	0.00797	0.00798	0.00799	0.00799
0.3	0.02700	0.02696	0.02697	0.02698	0.02698
0.4	0.06400	0.06396	0.06397	0.06398	0.06398
0.5	0.12500	0.12495	0.12497	0.12498	0.12498
0.6	0.21600	0.21596	0.21597	0.21598	0.21598
0.7	0.34300	0.34296	0.34298	0.34299	0.34298
0.8	0.51200	0.51197	0.51198	0.51199	0.51199
0.9	0.72900	0.72898	0.72899	0.72899	0.72899
**L**_∞_	…	4.04981 × 10^−5^	2.57172 × 10^−5^	1.22213 × 10^−5^	1.32122 × 10^−5^
**L**_**2**_	…	2.93710 × 10^−5^	1.86801 × 10^−5^	8.92226 × 10^−6^	9.48874 × 10^−6^

**Table 15:** Comparison of numerical solution and analytic solution of imaginary part with error norms for different values of *γ*, *N* = 150, $\Delta t=\frac{1}{1000}$ and 0 ≤ *ξ* ≤ 1 for 4.3.

*ξ*	$\hat{\phi }$	*ϕ*			
		*γ* = 0.8	*γ* = 0.85	*γ* = 0.9	*γ* = 0.95
0.1	0.99004	0.99005	0.99005	0.99005	0.99005
0.2	0.96078	0.96079	0.96079	0.96079	0.96079
0.3	0.91393	0.91393	0.91393	0.91393	0.91393
0.4	0.85214	0.85214	0.85215	0.85214	0.85214
0.5	0.77880	0.77880	0.77880	0.77880	0.77880
0.6	0.69767	0.69768	0.69768	0.69767	0.69767
0.7	0.61262	0.61263	0.61263	0.61262	0.61262
0.8	0.52729	0.52729	0.52729	0.52729	0.52729
0.9	0.44485	0.44485	0.44486	0.44485	0.44485
**L**_∞_	…	5.30461 × 10^−6^	6.59756 × 10^−6^	2.59029 × 10^−6^	2.29977 × 10^−6^
**L**_**2**_	…	3.69010 × 10^−6^	4.60805 × 10^−6^	1.71316 × 10^−6^	1.49236 × 10^−6^

**Table 16:** Errors with Δ*t* = 0.0005 of Example 4.3 at *t* = 1.

*ξ*	*γ* = 0.875, *N* = 200			*γ* = 0.875, *N* = 200		
	$\hat{\psi }$	*ψ*	Absolute Error	$\hat{\phi }$	*ϕ*	Absolute Error
0.1	0.00100	0.00099	3.68299 × 10^−6^	0.99005	0.99005	5.61085 × 10^−7^
0.2	0.00800	0.00799	6.38071 × 10^−6^	0.96078	0.96079	1.24183 × 10^−6^
0.3	0.02700	0.026991	8.13392 × 10^−6^	0.91393	0.91393	1.90892 × 10^−6^
0.4	0.06400	0.06399	9.00516 × 10^−6^	0.85214	0.85214	2.44449 × 10^−6^
0.5	0.12500	0.12499	9.07226 × 10^−6^	0.77880	0.77880	2.75635 × 10^−6^
0.6	0.21600	0.21599	8.41941 × 10^−6^	0.69767	0.69767	2.78454 × 10^−6^
0.7	0.34300	0.34299	7.12694 × 10^−6^	0.61262	0.61262	2.50317 × 10^−6^
0.8	0.51200	0.51199	5.26194 × 10^−6^	0.52729	0.52729	1.91844 × 10^−6^
0.9	0.72900	0.72899	2.87370 × 10^−6^	0.44485	0.44485	1.06452 × 10^−6^

**Table 17:** *L*_∞_(*N*) and *L*_2_(*N*) for different values of *γ* = 0.875 with $\Delta t=\frac{1}{2000}$, *N* = 100 and 0 ≤ *ξ* ≤ 1 of Example 4.3.

t	L_∞_(N)	*ϕ*	L_2_(N)	
	*ψ*		*ψ*	*ϕ*
0.1	1.60948 × 10^−5^	4.17642 × 10^−6^	1.16598 × 10^−5^	2.95700 × 10^−6^
0.5	1.31456 × 10^−5^	7.24164 × 10^−6^	9.51351 × 10^−6^	5.04977 × 10^−6^
0.9	8.92349 × 10^−6^	3.40613 × 10^−6^	6.49035 × 10^−6^	2.08787 × 10^−6^

## 6 Conclusion

This paper has presented a numerical strategy based on cubic B-spline functions (CBSFs) to efficiently solve the time fractional Schrödinger equation involving the Attangana-Baleanu time fractional derivative. The ABTFD was approximated using the conventional finite difference formulation, while CBSFs were utilised to interpolate the solution curve in the spatial direction. The current scheme in Table 11 exhibits higher precision compared to [27]. It is clear that the proposed approach demonstrated its novelty and achieved a satisfactory level of accuracy in the results. The proposed computational scheme demonstrated unconditional stability, and its effectiveness, simplicity, and adaptability were demonstrated through its implementation in numerical examples. Future research should expand the algorithm’s applications, analyse its properties, and explore its real-world applicability, examining its behaviour under different conditions and complex systems. The scheme’s utility and practicality could be enhanced by further investigation into its behavior under various boundary conditions and its performance in complex systems.

## Supporting information

S1 Dataset (PDF)
