# Supplementary material for: An efficient computational scheme for solving coupled time-fractional Schrödinger equation via cubic B-spline functions
Source: PLoS One. 2024 May 16;19(5):e0296909. doi: 10.1371/journal.pone.0296909 (PMC11098432; doi:10.1371/journal.pone.0296909)
Supplement: S1 Dataset — (PDF) [file pone.0296909.s001.pdf]

Minimal Data Set

Figures	Subfigure	Solution	t	Δt	N	γ	ξ
**Figure 1**	**(a)**	$\widehat{\psi}$	0.15,0.35,0.55,0.75,0.95	0.002	40	0.55	$\xi \in [0,1]$
	**(b)**	$\widehat{\phi}$	0.95,0.75,0.55,0.35	0.002	40	0.55	
**Figure 2**	**(a)**	$\widehat{\psi}$	1.0	0.001	100	0.55	$\xi \in [0,1]$
	**(b)**	$\widehat{\phi}$	1.0	0.001	100	0.55	
**Figure 3**	**(a)**	ψ	1.0	0.001	100	0.55	$\xi \in [0,1]$
	**(b)**	ϕ	1.0	0.001	100	0.55	
**Figure 4**	**(a)**	$\widehat{\psi}$	1.0	0.001	100	0.55	$\xi \in [0,1]$
	**(b)**	$\widehat{\phi}$	1.0	0.001	100	0.55	
**Figure 5**	**(a)**	$\widehat{\psi}$	0.95,0.75,0.65,0.45,0.25	0.002	40	0.15	$\xi \in [0,1]$
	**(b)**	$\widehat{\phi}$	0.95,0.75,0.65,0.45,0.25	0.002	40	01.5	
**Figure 6**	**(a)**	$\widehat{\psi}$	1.0	$\frac{1}{1000}$	100	0.55	$\xi \in [0,1]$
	**(b)**	$\widehat{\phi}$	1.0	$\frac{1}{1000}$	100	0.55	
**Figure 7**	**(a)**	ψ	1.0	$\frac{1}{1000}$	100	0.55	$\xi \in [0,1]$
	**(b)**	ϕ	1.0	$\frac{1}{1000}$	100	0.55	
**Figure 8**	**(a)**	$\widehat{\psi}$	1.0	$\frac{1}{1000}$	100	0.55	$\xi \in [0,1]$
	**(b)**	$\widehat{\phi}$	1.0	$\frac{1}{1000}$	100	0.55	
	**(a)**	$\widehat{\psi}$	1.0	$\frac{1}{1000}$	100	0.55	$\xi \in [0,1]$

**Figure 9**	**(b)**	$\widehat{\phi}$	1.0	$\frac{1}{1000}$	100	0.55	
**Figure 10**	**(a)**	$\widehat{\psi}$	1.0	$\frac{1}{1000}$	100	0.55	$\xi \in [0,1]$
	**(b)**	$\widehat{\phi}$	1.0	$\frac{1}{1000}$	100	0.55	
**Figure 11**	**(a)**	$\widehat{\psi}$	0.95,0.55,0.25	0.001	100	0.25	$\xi \in [0,1]$
	**(b)**	$\widehat{\phi}$	0.95,0.55,0.25	0.001	100	0.25	
**Figure 12**	**(a)**	$\widehat{\psi}$	1.0	0.001	100	0.25	$\xi \in [0,1]$
	**(b)**	$\widehat{\phi}$	1.0	0.001	100	0.25	
**Figure 13**	**(a)**	ψ	1.0	0.001	100	0.25	$\xi \in [0,1]$
	**(b)**	ϕ	1.0	0.001	100	0.25	
**Figure 14**	**(a)**	$\widehat{\psi}$	1.0	0.001	100	0.25	$\xi \in [0,1]$
	**(b)**	$\widehat{\phi}$	1.0	0.001	100	0.25	
**Figure 15**	**(a)**	$\widehat{\psi}$	1.0	0.001	100	0.25	$\xi \in [0,1]$
	**(b)**	$\widehat{\phi}$	1.0	0.001	100	0.25	
